# A Fur-regulated type VI secretion system contributes to oxidative stress resistance and virulence in *Yersinia pseudotuberculosis*

**DOI:** 10.1007/s44154-022-00081-y

**Published:** 2023-01-06

**Authors:** Yuxin Zuo, Changfu Li, Danyang Yu, Kenan Wang, Yuqi Liu, Zhiyan Wei, Yantao Yang, Yao Wang, Xihui Shen, Lingfang Zhu

**Affiliations:** State Key Laboratory of Crop Stress Biology for Arid Areas, Shaanxi Key Laboratory of Agricultural and Environmental Microbiology, College of Life Sciences, Northwest A&F University, Yangling, 712100 Shaanxi China

**Keywords:** Type VI secretion system, Fur, Manganese, Oxidative stress, Virulence

## Abstract

The type VI secretion system (T6SS) is a widespread protein secretion apparatus deployed by many Gram-negative bacterial species to interact with competitor bacteria, host organisms, and the environment. *Yersinia pseudotuberculosis* T6SS4 was recently reported to be involved in manganese acquisition; however, the underlying regulatory mechanism still remains unclear. In this study, we discovered that T6SS4 is regulated by ferric uptake regulator (Fur) in response to manganese ions (Mn^2+^), and this negative regulation of Fur was proceeded by specifically recognizing the promoter region of T6SS4 in *Y. pseudotuberculosis*. Furthermore, T6SS4 is induced by low Mn^2+^ and oxidative stress conditions via Fur, acting as a Mn^2+^-responsive transcriptional regulator to maintain intracellular manganese homeostasis, which plays important role in the transport of Mn^2+^ for survival under oxidative stress. Our results provide evidence that T6SS4 can enhance the oxidative stress resistance and virulence for *Y. pseudotuberculosis*. This study provides new insights into the regulation of T6SS4 via the Mn^2+^-dependent transcriptional regulator Fur, and expands our knowledge of the regulatory mechanisms and functions of T6SS from *Y. pseudotuberculosis*.

**Supplementary Information:**

The online version contains supplementary material available at 10.1007/s44154-022-00081-y.

## Introduction

Bacteria have evolved diverse protein secretion systems to transport individual proteins for interaction with the complex environment, bacterial competitors, and host organisms (Yu et al. 2021). The type VI secretion system (T6SS) is a widely deployed molecular weapon used by many Gram-negative bacteria to deliver effector proteins into target cells, with structural homology to the T4 bacteriophage tail spike and tube (Silverman et al. 2012). The functional T6SS consists of a minimal set of 14 core components, including TssA-M, Hcp, VgrG, and ClpV, that form the membrane complex, baseplate structure, and tail tube sheath complex, respectively (Cianfanelli et al. 2016; Shneider et al. 2013). Upon an unknown signal, the complex contractile nano-machine T6SS is immediately assembled to load different types of effectors, which is used to translocate effector proteins into target cells in a one-step manner (Basler et al. 2012; Russell et al. 2011). Thus, T6SS is an important and versatile system with diverse functions in interbacterial competition, antifungal activity, virulence, and stress responses, and has been carried out by targeting various proteins in bacteria, fungi, host, and other organisms (Ho et al. 2017; Trunk et al. 2018; Wang et al. 2015; Xu et al. 2014a).

The expression and function of T6SS as a bacterial protein translocation machine are highly energy-intensive processes, so it needs to be tightly regulated. Recent studies have demonstrated various regulators specifically modulate T6SS activity in response to environmental cues that regulate bacterial survival, competition, and pathogenesis under harsh conditions. For example, T6SS in *Vibrio cholerae* is activated by the osmoregulator OscR and the cold-shock protein CspV to cope with high osmotic pressure and elevated temperature (Ishikawa et al. 2012; Townsley et al. 2016). In *Burkholderia thailandensis*, T6SS is significantly activated by the oxidative stress regulator OxyR and zinc (Zn) uptake regulator Zur, which are involved in oxidative stress resistance and contact-independent interbacterial competition (Si et al. 2017a; Si et al. 2017b). In *Pseudomonas aeruginosa* and *Cupriavidus pinatubonensis*, T6SSs are regulated by iron (Fe) availability through the repression of ferric uptake regulator Fur. Under iron-restricted conditions, Fur-repressed T6SSs are de-repressed and secrete the effectors TseF and TeoL to recruit outer membrane vesicles (OMVs) for iron acquisition, thereby performing pleiotropic physiological functions (Li et al. 2022; Lin et al. 2017). Together, these regulatory mechanisms provide insights into how bacteria coordinate T6SS activity under multiple adverse environmental conditions.

*Yersinia pseudotuberculosis* is an enteric Gram-negative pathogen that is widely distributed in the environment; it is transmitted to mammalian hosts following the consumption of contaminated food or water, and causes a wide range of gastrointestinal diseases including appendicitis, ileitis, colitis, and mesenteric lymphadenitis (Lamps et al. 2003; Weber et al. 1970). *Y. pseudotuberculosis* harbours four types of T6SS gene clusters with multiple functions, and has become a model organism for studying the function and regulation of T6SS (Zhang et al. 2011). The expression of T6SS1 is induced at 37 °C in *Y. pseudotuberculosis*, suggesting that it may play a more important role in bacterial virulence during host infection than other T6SSs (Zhang et al. 2011). In mice, *Y. pseudotuberculosis* T6SS3 mediates contact-dependent mortality through the injection of the nuclease effector Tce1 into adjacent cells, which facilitates gut colonization, thereby improving bacterial survival and competitive advantage (Song et al. 2021). T6SS4 in *Y. pseudotuberculosis* was reported to be regulated by OmpR, RovM, OxyR, and Zur, which was in relation to metal ion uptake and various environmental stresses resistance (Cai et al. 2021; Gueguen et al. 2013; Song et al. 2015; Wang et al. 2015; Zhang et al. 2011). Recently, we reported that *Y. pseudotuberculosis* T6SS4 is involved in manganese (Mn) acquisition through secretion of a Mn^2+^-binding micropeptide TssS, which is delivered into host cells to inhibit a STING-mediated innate immune response by sequestering Mn^2+^ (Zhu et al. 2021). However, the understanding of the roles of T6SSs in *Y. pseudotuberculosis* remains incomplete, and their functions and regulation networks require further exploration.

In this study, we found that the expression of T6SS4 is significantly repressed by Fur in *Y. pseudotuberculosis* YPIII, and further studies showed that Fur negatively regulates T6SS4 in a Mn^2+^-dependent manner by directly binding to the T6SS4 promoter region. Interestingly, the Fur-mediated repression of T6SS4 expression is eliminated upon oxidative challenge, and T6SS4 plays an important role in the transport of Mn^2+^ for bacterial survival. Our results demonstrated that T6SS4 not only contributes to combating oxidative stress in *Y. pseudotuberculosis* but also enhances the virulence by promoting the colonization of bacteria in infecting mice.

## Results

### Analysis of differentially expressed genes (DEGs) related to T6SSs regulated by Fur in *Y. pseudotuberculosis*

**Fig. 1:**
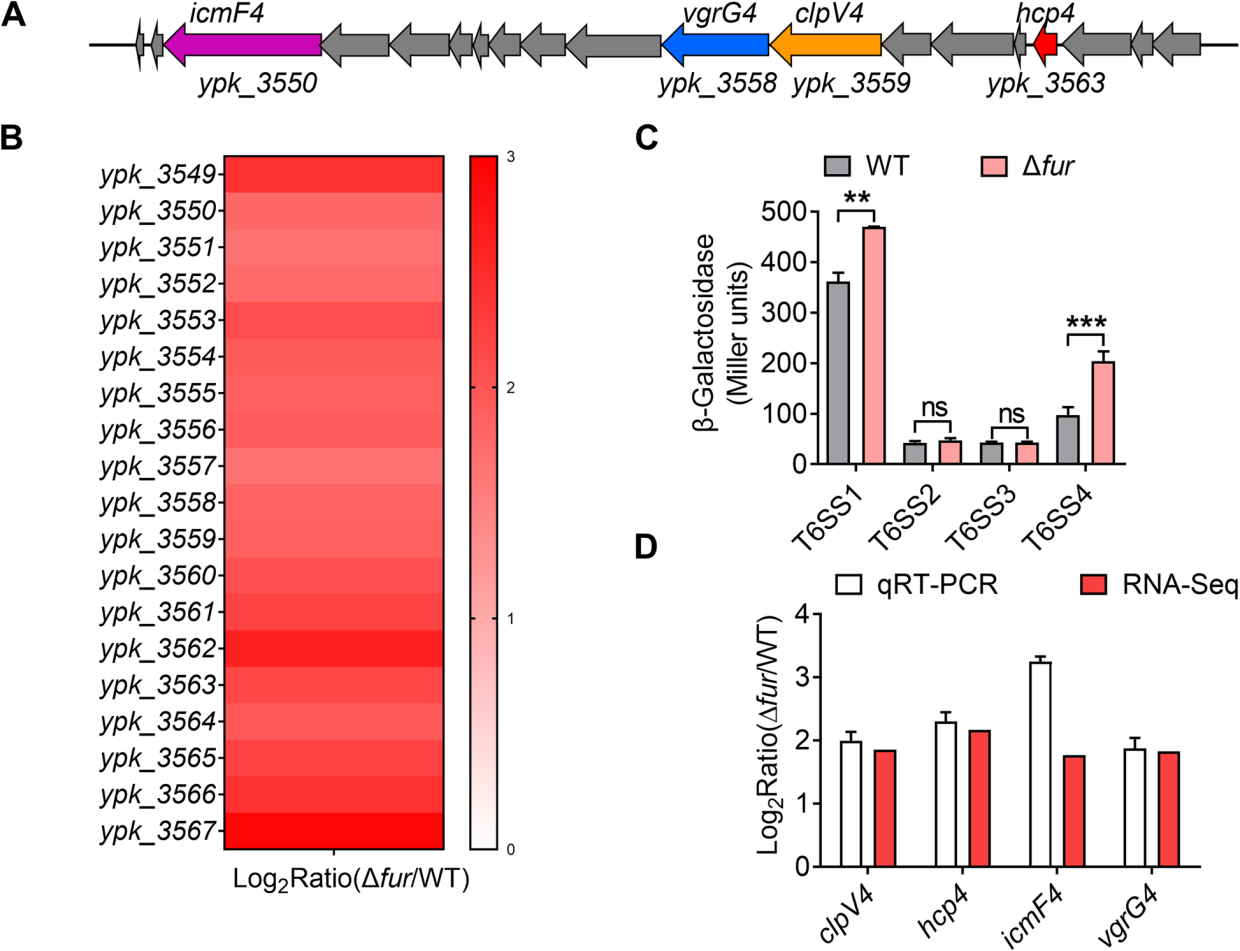
Transcriptomic analysis of Fur regulated T6SS genes in *Y. pseudotuberculosis*. **A** Gene organization of T6SS4 gene cluster in *Y. pseudotuberculosis*. **B** Heat map of transcriptomic analysis. All genes in the T6SS4 differentially transcribed in the *Y. pseudotuberculosis* Δ*fur* mutant compared with those in the WT were detected by RNA-seq data analysis. **C** β-galactosidase assay analysis of T6SS1–4 expression levels in *Y. pseudotuberculosis* WT and Δ*fur* mutant strains grown to stationary phase in YLB medium. **D** qRT-PCR analysis of T6SS4 expression level. Genes differentially transcribed in *Y. pseudotuberculosis* Δ*fur* mutant compared with those in the WT were detected by transcriptomic and qRT-PCR analysis. Four representative genes (*clpV4*, *hcp4*, *icmF4*, and *vgrG4*) were chosen to validate the RNA-seq data by qRT-PCR. The white bars represent the mean values obtained for the reference WT and three biological replicates. Error bars indicate the SEM. Red bars represent RNA-seq data. Data represent the mean ± SEM of three biological replicates, each of which was performed with three technical replicates. **P < 0.01; ***P < 0.001; ns, not significant

As reported that Fur plays important physiological roles in ion homeostasis (such as iron and manganese), oxidative stress response, and full virulence (Askoura et al. 2016; Hohle and O'Brian 2016; Troxell and Hassan 2013), and can directly or indirectly regulate T6SSs in various pathogens (Brunet et al. 2011; Sana et al. 2012; Storey et al. 2020; Wang et al. 2019). Genomic analyses revealed a Fur ortholog (*ypk*_*2991*) in *Y. pseudotuberculosis*, and the protein sequence shares 86%, 56%, 78%, 99%, and 50% similarity (Fig. S1) with previously identified Fur in *E. coli*, *P. aeruginosa*, *Vibrio fischeri*, *Yersinia pestis*, and *C. pinatubonensis*, respectively (Li et al. 2019; Pasqua et al. 2017; Seo et al. 2014; Septer et al. 2013; Zhou et al. 2006). To determine whether Fur regulates T6SSs (Fig. S2) in *Y. pseudotuberculosis*, we performed RNA sequencing (RNA-seq)-based transcriptomic analysis and identified DEGs related to T6SSs, and screened using a threshold of |log_2_Ratio(Δ*fur*/WT)| ≥ 1. Only entire T6SS4 (Fig. 1A) genes showed significantly enhanced transcription in the Δ*fur* mutant compared to those in the wild type (WT) (Fig. 1B), but not T6SS1, T6SS2, and T6SS3 (Fig. S3A-C). Entire T6SS1 genes just showed slightly enhanced transcription (0.185 ≤ log_2_Ratio(Δ*fur*/WT) ≤ 2.160) (Fig. S3A). In addition, the promoter activity of T6SS1–4 in *Y. pseudotuberculosis* WT and Δ*fur* was also determined. The result suggested that deletion of *fur* significantly increased the expression of T6SS1 and T6SS4, particularly T6SS4, whereas the expression of T6SS2 or T6SS3 was little affected in the Δ*fur* mutant (Fig. 1C). Next, we validated the transcriptomic data using quantitative real-time polymerase chain reaction (qRT-PCR) analysis of the *clpV4*, *hcp4*, *icmF4*, and *vgrG4* genes, which are the main components of T6SS4. Consistent with the RNA-seq data, the expression of these genes was upregulated in the Δ*fur* mutant (Fig. 1D). Thus, these data suggest that Fur significantly represses the expression of entire genes in T6SS4.

### Fur negatively regulates T6SS4 expression by directly binding to its promoter

**Fig. 2:**
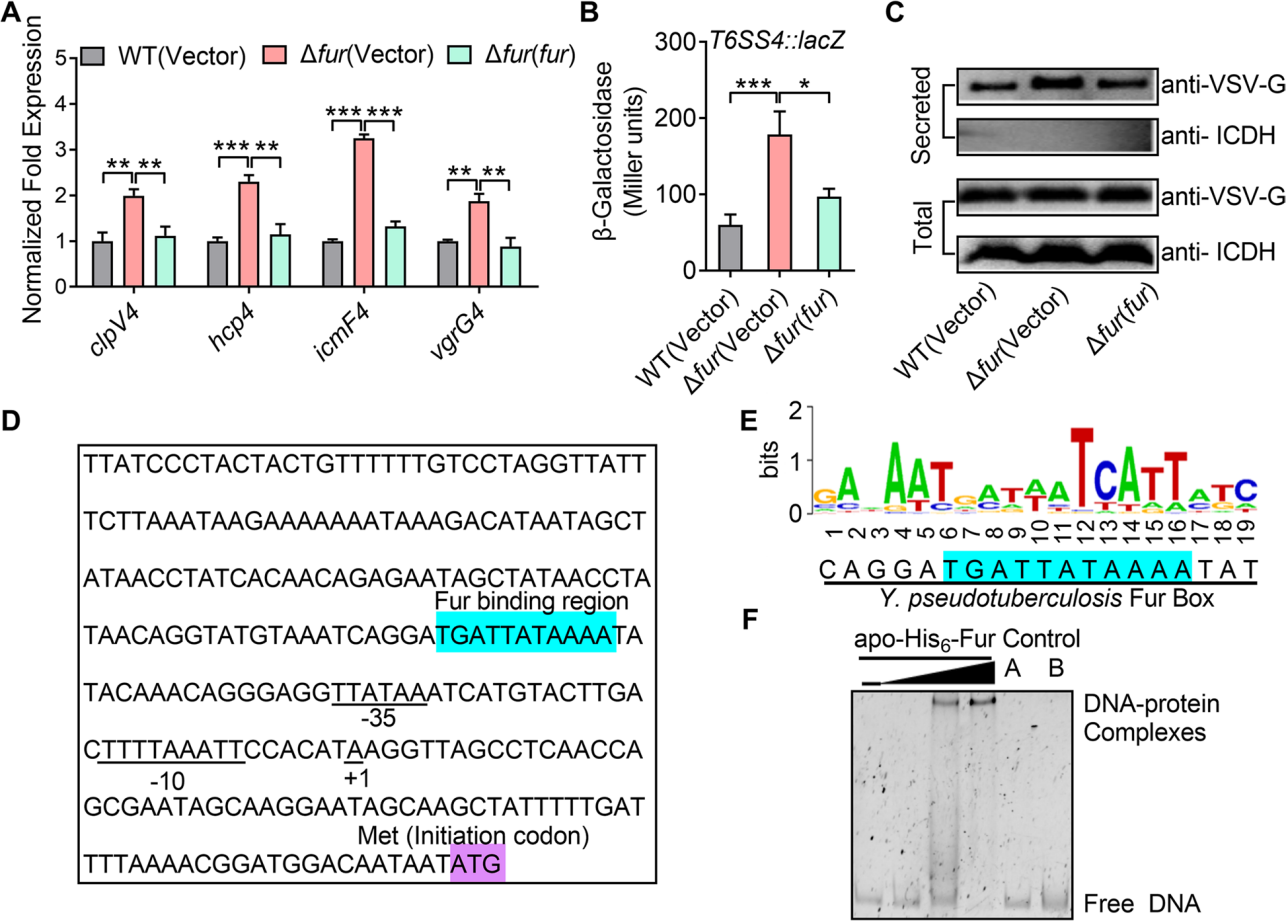
Fur represses the expression of T6SS4 by directly binding to its promoter. **A** qRT-PCR analysis of mRNA levels of T6SS4. Cells of relevant *Y. pseudotuberculosis* strains were grown to mid-exponential phase in YLB medium and the expression of *clpV4*, *hcp4*, *icmF4*, and *vgrG4* (the main components of T6SS4) was measured by qRT-PCR. **B** β-galactosidase analysis of T6SS4 promoter activity was performed by using the transcriptional *P*_*T6SS4*_*::lacZ* chromosomal fusion reporter expressed in indicated bacterial strains grown to stationary phase in YLB medium. **C** Protein levels of Hcp4 in relevant strains. These related strains expressing C-terminal VSV-G-tagged Hcp4 were grown in YLB medium to the late logarithmic phase at 26 °C. Expression (Total) and secretion (Secreted) of Hcp4-VSV-G were detected by immunoblotting using anti-VSV-G antibodies. Isocitrate dehydrogenase (ICDH) was used as a loading control. **D** Identification of the Fur binding site in the promoter region of T6SS4. Putative Fur binding site was identified in blue by the online software Virtual Footprint. The ATG start codon of the first ORF of the T6SS4 operon was marked in purple, and the − 35 and − 10 elements of the T6SS4 promoter are underlined. + 1 denotes the transcription start point. **E** Fur box sequence upstream of *ypk*_*3566*. Virtual footprint analysis of the *Y. pseudotuberculosis* Fur binding sequence. Letters represent position weight matrix based on *P. aeruginosa* consensus sequence for Fur binding. The Y-axis represents relative nucleotide probability and the X-axis represents nucleotide position. *Y. pseudotuberculosis* Fur box sequence is located at − 134 bp of *ypk*_*3566* and has a probability score of 9.26 (max score = 11.32). **F** EMSA was performed to analyze the interaction between His_6_-Fur and the T6SS4 promoter (*P*_*T6SS4*_) in the presence 100 μM Mn^**2**+^. Increasing amounts of Fur (0.12, 0.24, and 0.96 μM) and 4 ng DNA fragment were used (Control A, unrelated DNA fragment; Control B, BSA). Data represent the mean ± SEM of three biological replicates, each of which was performed with three technical replicates. *P < 0.05; **P < 0.01; ***P < 0.001

T6SS4 in *Y. pseudotuberculosis* has been reported to be regulated by OmpR, which is necessary for bacteria to combat acid stress through maintaining intracellular pH homeostasis (Zhang et al. 2013). To further explore the functions and regulatory mechanisms of T6SS4, we determined the negative regulation of Fur on T6SS4 by using qRT-PCR analysis. From the result, the expression level of *clpV4*, *hcp4*, *icmF4*, and *vgrG4* genes was significantly increased in the Δ*fur* mutant, and this increase was completely reversed by a complementation plasmid expressing the regulatory protein Fur (Fig. 2A). We also determined its negative regulation on T6SS4 by measuring the transcription activity of chromosomal *P*_*T6SS4*_::*lacZ* fusions. The T6SS4 promoter activity was significantly increased in the Δ*fur* mutant, which could be fully restored in the complemented strain Δ*fur*(*fur*) (Fig. 2B). The difference above was not attributable to the growth defect, as all strains adopted in the assay grew equally in Yersinia–Luria–Bertani (YLB) medium (Fig. S4). What’s more, we found the secretion of Hcp4 was increased in the Δ*fur* mutant (Fig. 2C), which further confirmed the negative regulation of Fur. Taken together, these results indicate that Fur negatively regulates T6SS4 expression in *Y. pseudotuberculosis*.

We further analyzed the promoter region of T6SS4 in *Y. pseudotuberculosis*, and revealed a putative Fur-binding site (TGATTATAAAA) (Fig. 2D), which was highly similar to the Fur box in *P. aeruginosa* (Fig. 2E). Fur has been reported to act as an iron- and manganese-responsive regulator in *S. typhimurium* and *Y. pestis (*Bearden and Perry 1999*;* Kehres et al. 2002*)*. To determine whether Fur regulates T6SS4 expression directly in a metal-dependent manner in *Y. pseudotuberculosis*. First, we performed metal ion binding assay, and found Fur can directly interact with Mn^2+^ and Fe^2+^ (Fig. S5A). Next, to examine the interaction between Fur and the T6SS4 promoter, the metal free apo-Fur protein (Fig. S6) and a probe containing the T6SS4 promoter (*P*_*T6SS4*_) sequence [− 1 to − 350 relative to the ATG start codon of the first open reading frame of the T6SS4 operon] were incubated in the presence or absence of 100 μM Fe^2+^ or 100 μM Mn^2+^, which were then analyzed by electrophoretic mobility shift assay (EMSA). No protein-DNA complexes were observed in the absence of metal in the binding reaction, but Fur bound the promoter in the presence of Fe^2+^ or Mn^2+^, showing that the binding is metal-dependent (Fig. S5B and Fig. 2F). And the result suggested that Mn^2+^ was more efficient to increase the binding activity between Fur and T6SS4 promoter. Meanwhile, the specific interaction was also confirmed as excessive unrelated probe and protein failed to form the protein-DNA complexes (Fig. 2F). Thus, we conclude that Fur negatively regulates T6SS4 expression by specifically recognizing an operator within the T6SS4 promoter region in a Mn^2+^-responsive manner.

### Fur-mediated expression of T6SS4 responds to Mn^2+^ concentration

**Fig. 3:**
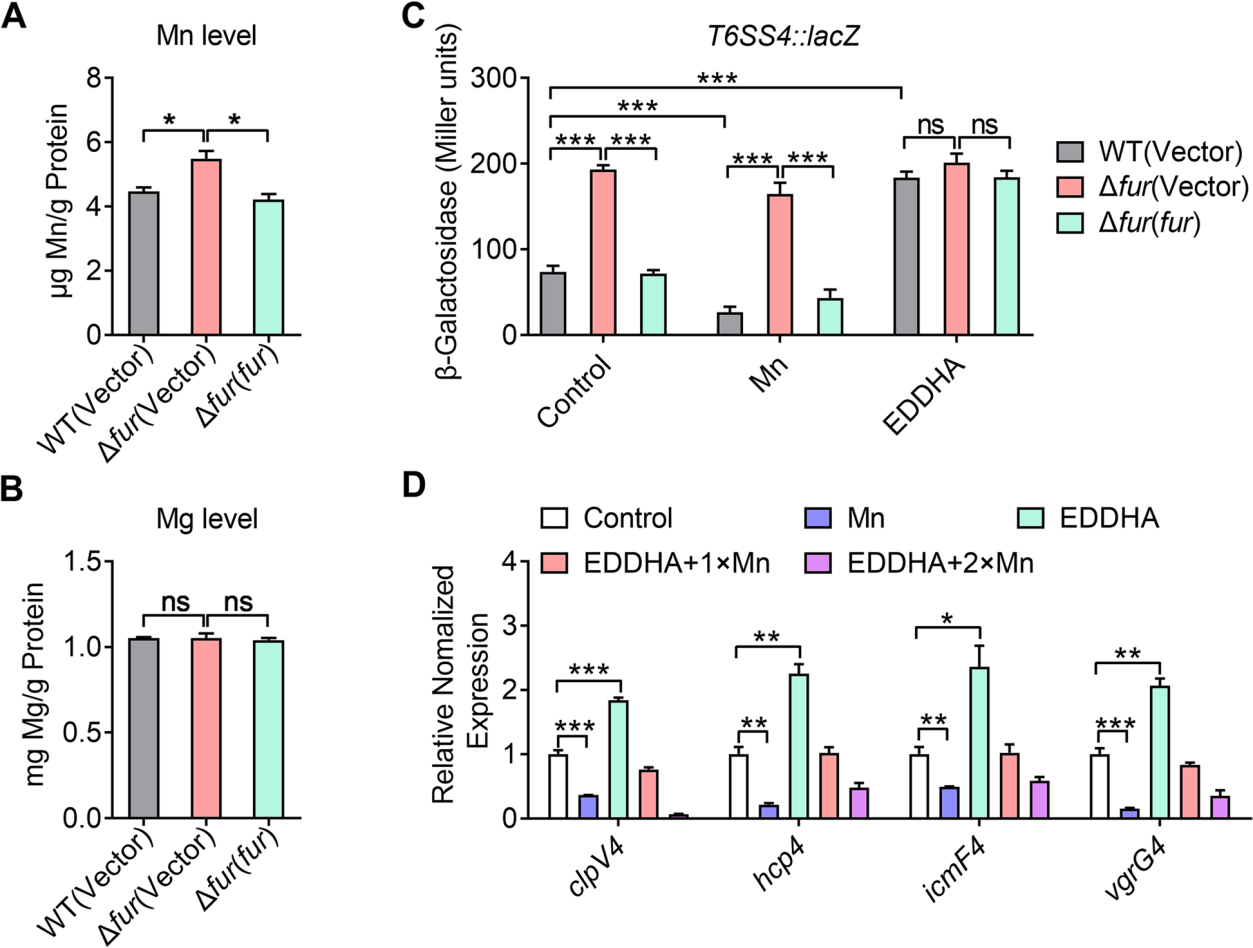
Fur-mediated expression of T6SS4 responds to Mn^**2**+^ concentration. **A-B** The Δ*fur* mutant accumulates intracellular Mn^**2**+^. Intracellular manganese and magnesium were measured by ICP-MS in the *Y. pseudotuberculosis* strains grown to the end of logarithmic phase in YLB medium. **C** Fur-mediated expression of T6SS4 responds to different Mn^**2**+^ concentrations. β-galactosidase analysis of T6SS4 promoter activities was performed in *Y. pseudotuberculosis* strains grown to stationary phase in YLB medium with or without 100 μM EDDHA and 100 μM Mn^**2**+^. **D** T6SS4 expression is induced by low Mn^**2**+^ conditions. qRT-PCR analysis of mRNA levels of T6SS4 in the *Y. pseudotuberculosis* WT strain grown to mid-exponential phase in YLB medium containing 100 μΜ Mn^**2**+^, 100 μΜ EDDHA, 100 μΜ EDDHA with 100 μΜ Mn^**2**+^ (EDDHA + 1× Mn^**2**+^), or 100 μΜ EDDHA with 200 μΜ Mn^**2**+^ (EDDHA + 2× Mn^**2**+^). Data represent the mean ± SEM of three biological replicates, each of which was performed with three technical replicates. *P < 0.05; **P < 0.01; ***P < 0.001; ns, not significant

The above data showed that Fur directly represses T6SS4 expression (Fig. 2). Noteworthily, our previous studies have reported that T6SS4 is involved in the Mn^2+^ transport by secreting Mn^2+^-binding protein TssS, and Fur is a global regulator of iron homeostasis in *Y. pseudotuberculosis (*Li et al. 2021*;* Zhu et al. 2021*)*. To investigate whether Fur plays a role in manganese metabolism via the regulation of Mn^2+^ transport pathways, such as T6SS4 in *Y. pseudotuberculosis*, we measured the total metal content in bacterial cells using inductively coupled plasmon resonance atomic absorption spectrometry (ICP-MS). The results showed that the Δ*fur* mutant accumulated more intracellular manganese than the WT and Δ*fur*(*fur*) complemented strains (Fig. 3A). By contrast, deletion of the *fur* gene had little effect on the accumulation of magnesium (Mg) (Fig. 3B). Thus, Fur is required for *Y. pseudotuberculosis* to maintain manganese homeostasis.

To determine whether Fur-mediated negative regulation of T6SS4 responds to Mn^2+^ concentration in *Y. pseudotuberculosis*, we determined the expression of T6SS4 in WT strain using chromosomal *P*_*T6SS4*_*::lacZ* fusion reporter analysis at different Mn^2+^ concentrations. As shown in Fig. 3C, the addition of exogenous Mn^2+^ inhibited T6SS4 expression, while ethylenediamine-*N,N*′-bis(2-hydroxyphenylacetic acid) (EDDHA), a Mn^2+^ chelator, activated it; this activation was reversed with the addition of Mn^2+^, suggesting that T6SS4 expression was responsive to the level of Mn^2+^ in the environment. We also compared the expression of T6SS4 between the WT and Δ*fur* mutant strains, and found that the Δ*fur* mutant showed a significantly increased promoter activity compared to the WT under manganese-rich conditions, while no expression difference was detected under manganese-limited conditions, indicating that Fur normally negatively affected T6SS4 expression in the presence of Mn^2+^ (Fig. 3C). Regulation of the T6SS4 operon by Mn^2+^ was further confirmed using qRT-PCR analysis, showing that the expression of T6SS4 core component genes, such as *vgrG4*, *clpV4*, *icmF4*, and *hcp4*, was induced at low Mn^2+^ concentrations and repressed by exogenous Mn^2+^ in a concentration-dependent manner (Fig. 3D). Altogether, these data above collectively indicate that the negative regulation of T6SS4 operon by Fur is Mn^2+^ concentration-dependent in *Y. pseudotuberculosis*.

### Fur-regulated T6SS4 combats oxidative stress by importing Mn^2+^

**Fig. 4:**
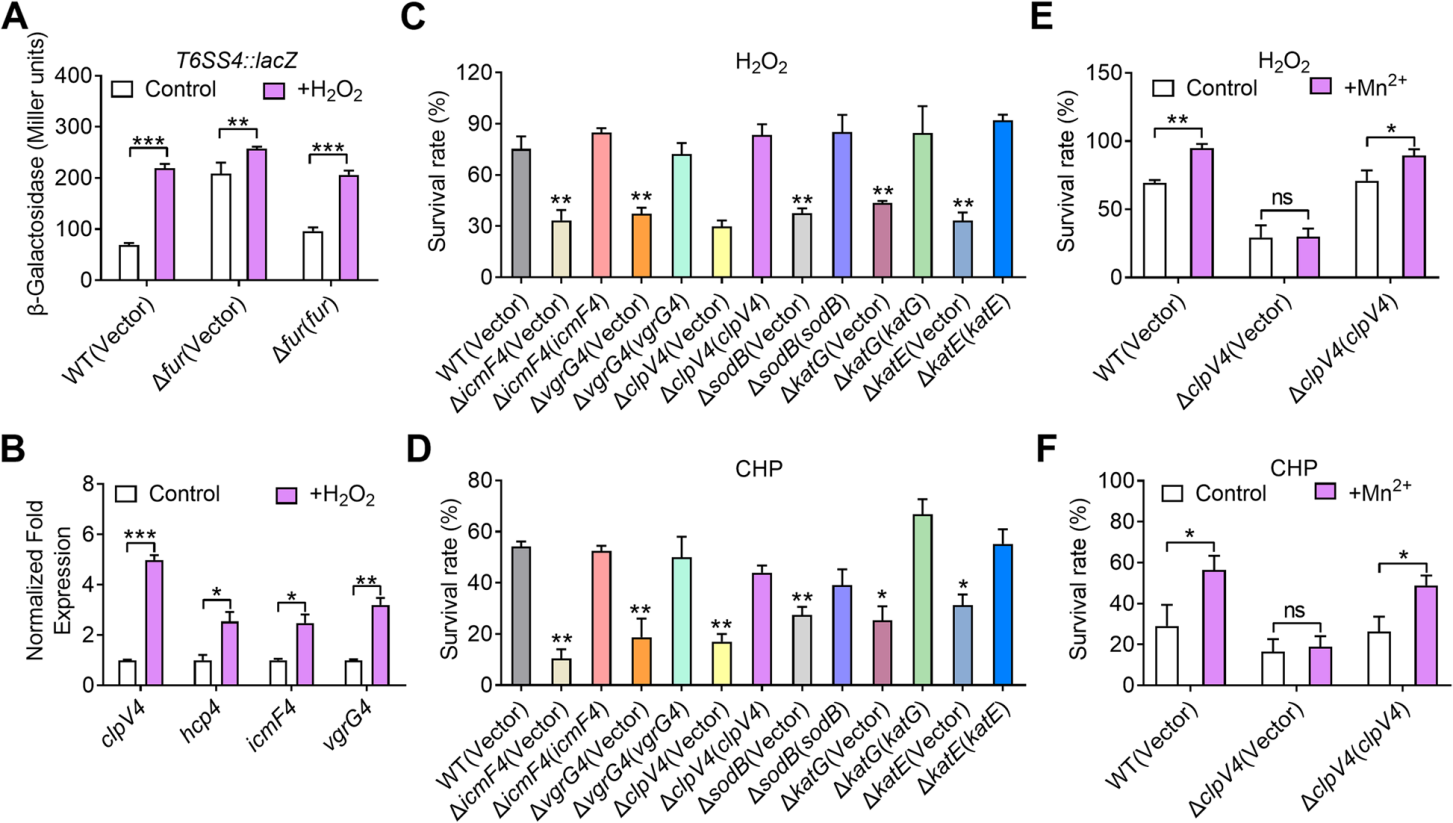
Fur-regulated T6SS4 combats oxidative stress by importing Mn^**2**+^. **A** The negative regulation of T6SS4 by Fur was derepressed by H_2_O_2_ challenge. β-galactosidase analyses of T6SS4 promoter activities in *Y. pseudotuberculosis* strains grown to stationary phase in YLB medium with or without 5 mM H_2_O_2_. **B** T6SS4 expression responds to oxidative stress. *Y. pseudotuberculosis* WT was grown in M9G medium with or without 5 mM H_2_O_2_, and the expression of *clpV4*, *hcp4*, *icmF4*, and *vgrG4* was measured by qRT-PCR. **C-D** T6SS4 is involved in oxidative stress resistance. The viability of mid-exponential phase *Y. pseudotuberculosis* strains was determined after exposure to 1 mM H_2_O_2_ or 0.5 mM CHP for 40 min in M9G medium. **E-F** The alleviation of the sensitivity of *Y. pseudotuberculosis* strains to oxidative stress by exogenous Mn^**2**+^ required T6SS4. Relevant mid-exponential phase bacterial strains were exposed to 1 mM H_2_O_2_ or 0.5 mM CHP in M9G medium with or without exogenously provided Mn^**2**+^ (1 μM) for 40 min and the viability of the cells was determined. Data represent the mean ± SEM of three biological replicates, each of which was performed with three technical replicates. *P < 0.05; **P < 0.01; ***P < 0.001; ns, not significant

As mentioned above, *Y. pseudotuberculosis* T6SS4-mediated Mn^2+^ transport is under Fur control in an Mn^2+^-dependent manner. Mn^2+^ plays a crucial role in protecting against oxidative damage. Hydrogen peroxide (H_2_O_2_) is an endogenous reactive species that is used as an over-the-counter antiseptic against bacteria (Murphy and Friedman 2019). To verify whether Fur-regulated T6SS4 expression responds to H_2_O_2_, we investigated the expression of T6SS4 in the WT, Δ*fur* mutant, and complemented Δ*fur*(*fur*) strains by measuring the transcription of chromosomal *P*_*T6SS4*_::*lacZ* fusions following H_2_O_2_ challenge. Interesting, T6SS4 expression was significantly induced by the addition of 5 mM H_2_O_2_, and Fur-mediated repression of T6SS4 was almost eliminated upon H_2_O_2_ challenge (Fig. 4A). Furthermore, the effect of H_2_O_2_ on T6SS4 expression was further confirmed based on qRT-PCR analysis, which showed that the expression of *clpV4*, *hcp4*, *icmF4*, and *vgrG4* was enhanced by H_2_O_2_ (Fig. 4B). Thus, oxidative stress relieves Fur-mediated repression of T6SS4 transcription.

To further verify the effects of T6SS4 on bacterial resistance to oxidative stress in *Y. pseudotuberculosis*, we determined the viability of T6SS4 mutants following challenges with oxidative stressors such as H_2_O_2_ and cumene hydroperoxide (CHP) (Rider et al. 2016). The survival rates of mutants Δ*icmF4*, Δ*vgrG4*, and Δ*clpV4*, which lack conserved T6SS4 structural genes, were significantly decreased compared to the WT (Fig. 4C and D). The genes *sodB*, *katG*, and *katE* were used as controls because they are crucial for the protection of bacteria from oxidative stress (Kim and Yu 2012; Ruiz-Laguna et al. 2000; Vaze et al. 2017; Zhu et al. 2019), and they were all significantly more sensitive to H_2_O_2_ and CHP than WT strain (Fig. 4C and D). In addition, the survival rates of all complemented strains were almost completely restored to the WT levels (Fig. 4C and D), further supporting the role of T6SS4 in combating oxidative stress. To investigate whether the antioxidant function of *Y. pseudotuberculosis* T6SS4 is related to Mn^2+^ acquisition, we determined the survival rate of *Y. pseudotuberculosis* relevant strains under oxidative stress by supplying with exogenous Mn^2+^(1 μM) or not. The result showed that Mn^2+^ markedly protected the WT and complemented strain Δ*clpV4*(*clpV4*) from CHP challenge, while failing in the Δ*clpV4* mutant (Fig. 4E and F), indicating that T6SS4 in *Y. pseudotuberculosis* can import Mn^2+^ to combat oxidative stress. Collectively, the data above demonstrate that the *Y. pseudotuberculosis* T6SS4 is effectively induced under oxidative stress, and thus plays an important role in transporting Mn^2+^ for bacterial survival.

### T6SS4 enhances the virulence of *Y. pseudotuberculosis* in infected mice

**Fig. 5:**
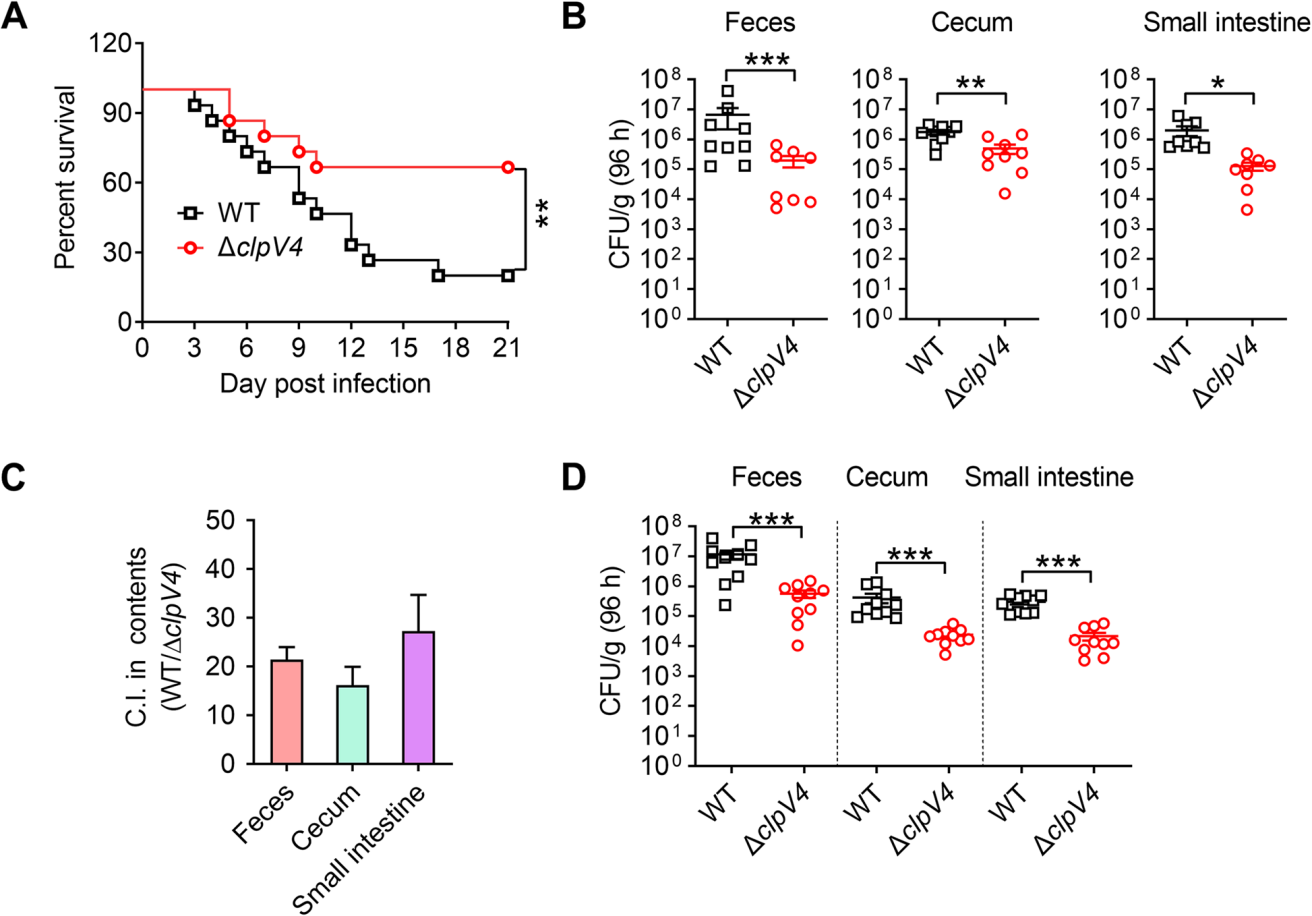
T6SS4 enhances the virulence and colonization *o*f *Y. pseudotuberculosis* in infected mice. **A-B***Y. pseudotuberculosis* WT and Δ*clpV4* (the main components of T6SS4) mutant strains grown in YLB were washed twice in sterilized PBS and used for orogastric infection of 6–7 weeks old female C57BL/6 mice using a ball-tipped feeding needle. For survival assays, 1 × 10^9^ bacterial cells of each strain were applied to different groups of mice (*n* = 15/strain), and the survival rate of the mice was determined (**A**). Enumeration of bacterial burdens in the feces, cecum, and small intestine (**B**) of infected C57BL/6 mice at 4 days post-infection by CFU assays (*n* = 8–9). **C-D** 6–7 weeks old female C57BL/6 mice were coinfected 1:1 with *Y. pseudotuberculosis* WT and Δ*clpV4* (the main components of T6SS4) mutant strains (*n* = 10/group). index (C.I.) = output ratio (CFU *Y. pseudotuberculosis* WT/Δ*clpV4*) divided by input ratio. Bars denote mean C.I. ± SEM (**C**). Enumeration of *Y. pseudotuberculosis* WT and Δ*clpV4* strain burdens in the feces, cecum, and small intestine of infected C57BL/6 mice at 3 days post-infection by CFU assays (n = 10) (**D**). Similar results were obtained in three independent experiments, and data shown are from one representative experiment done in triplicate. Statistical analysis was performed by Log-Rank test (**A**). The statistical significance was determined by the Mann-Whitney test (**B** and **D**). *P < 0.05; **P < 0.01; ***P < 0.001

T6SSs in many pathogenic bacteria have been reported to play important roles in implementing virulence (Miyata et al. 2011; Mougous et al. 2006; Schell et al. 2007). To investigate whether T6SS4 contributes to the virulence of *Y. pseudotuberculosis*, we determined the survival rates of C57BL/6 mice after intragastrically infected with the WT or Δ*clpV4* strain, and found that the survival of mice was less than 20% within 3 weeks after inoculation with WT strain, while mice infected with Δ*clpV4* mutant lacking T6SS4 survived better (Fig. 5A). In addition to acting as a virulence factor in many pathogenic bacteria, T6SS is equally important for its survival in the host (Anderson et al. 2017; Hsieh et al. 2019; Kapitein and Mogk 2013; Koskiniemi et al. 2013; Sana et al. 2016). To explore whether *Y. pseudotuberculosis* T6SS4 plays a role in survival and can well adapt to multiple microbiotas of host environment. We determined the bacterial loads recovered from the feces, cecum, and small intestine after infected with the relevant *Y. pseudotuberculosis* strains for 96 h, and found mice infected with Δ*clpV4* mutant had significantly lower bacterial loads compared to WT-infected mice (Fig. 5B). Furthermore, mice were co-infected with a 1:1 mixture of *Y. pseudotuberculosis* WT and the T6SS4 mutant Δ*clpV4* for 96 h, and then feces and related tissues were extracted for colony-forming unit (CFU) analysis. After co-infection, the ratios increased to approximately 21:1, 16:1, and 27:1, in the feces, cecum, and small intestine respectively (Fig. 5C and D), indicating that the WT was more fitness in the tissues of infected mice than Δ*clpV4* mutant.

**Fig. 6:**
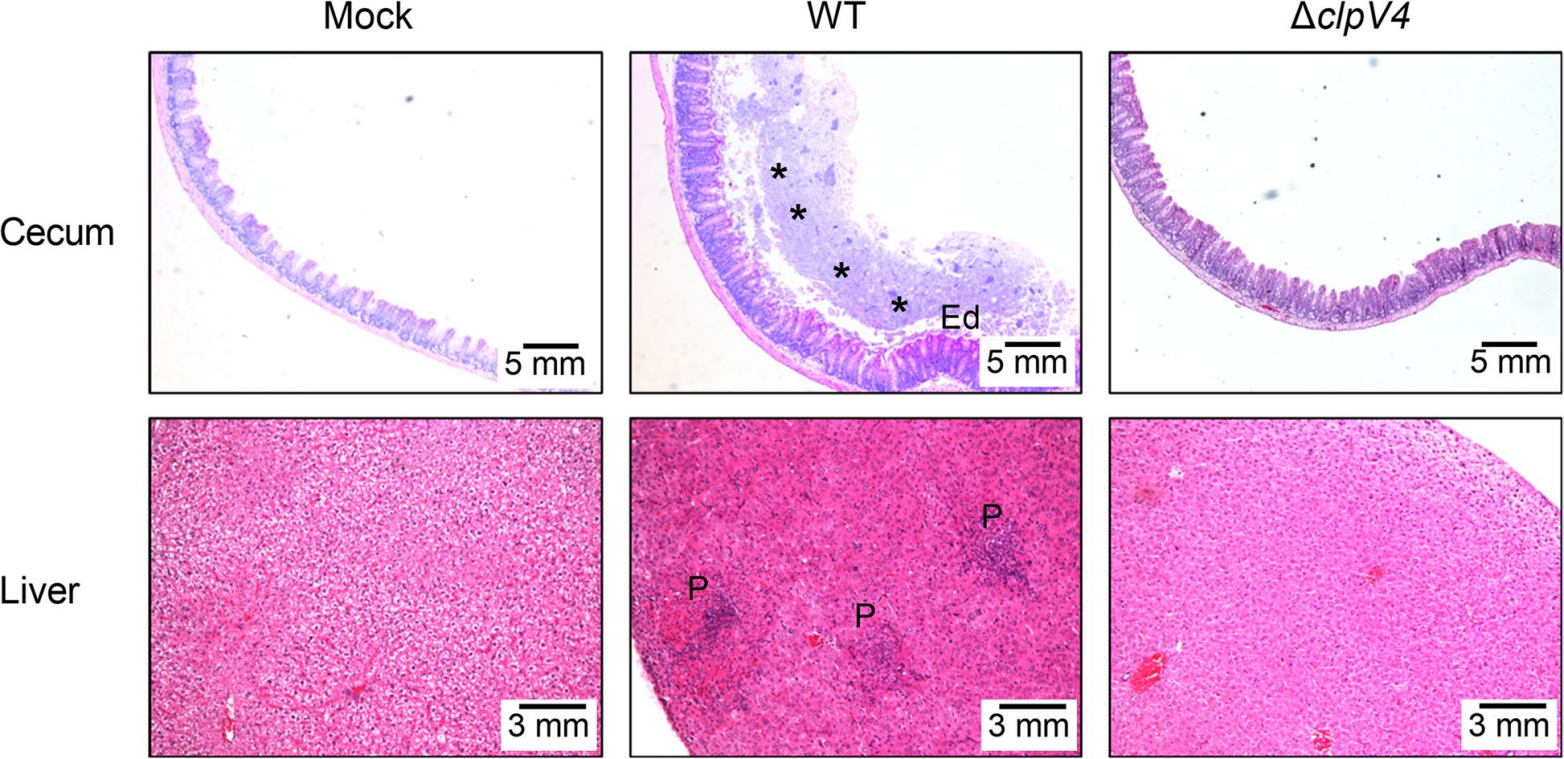
Histopathological changes in the cecum and liver of infected mice. Hematoxylin-Eosin (H&E) staining of the cecum and liver of the C57BL/6 mice intragastrically inoculated with *Y. pseudotuberculosis* WT or Δ*clpV4* mutant. Tissues were collected 48 h post infection. Pictures show representatives of multiple fields of sections from groups of 3–5 mice. Dashed halo (*), focal invasion of lymphocytes into the lamina propria; Ed: edema formation. P: piecemeal necrosis

The virulence was also assessed by comparing the histopathological changes in the cecum and liver of infected and control mice. After staining with hematoxylin and eosin (H&E), pathological changes were examined under a light microscope. In the cecum, infection with the WT caused more severe inflammation than that with the Δ*clpV4* mutant during the early acute phase (2 dpi); the inflammation was diffuse and affected the entire lamina propria and the cecal lymphoid follicles (Fig. 6). Because *Y. pseudotuberculosis* infection spreads systemically to reach the liver (Schweer et al. 2013), we further conducted histopathological examination of infected livers and found that piecemeal necrosis was more generalized in the WT- than in Δ*clpV4*-infected livers (Fig. 6). These results indicate that *Y. pseudotuberculosis* T6SS4 leads to more severe and widespread inflammation in the cecum and liver of infected mice. Taken together, these results above establish that T6SS4 contributes to the virulence and enhances the survival of *Y. pseudotuberculosis* in infected mice.

## Discussion

The widely distributed T6SS is a multi-functional weapon deployed by Gram-negative bacteria, contributing not only to virulence and competition by targeting host cells or rival bacteria, but also responses to external stresses. Most importantly, the elaboration and usage of T6SS are energetically costly, such that bacteria have evolved regulatory systems to make it highly controlled and efficient in coping with adverse environments. Therefore, understanding these regulatory networks and their corresponding responses is the focus of substantial T6SS research. In this study, we demonstrate that T6SS4 from *Y. pseudotuberculosis* is negatively regulated by Fur in a Mn^2+^-dependent manner, which functions to combat oxidative stress by importing Mn^2+^, and that it plays crucial roles in virulence and survival in infected mice.

We demonstrated that Fur mediates Mn^2+^-dependent expression of *Y. pseudotuberculosis* T6SS4 (Fig. 3). It also controls T6SS expression in *E. coli*, *Edwardsiella tarda*, *P. aeruginosa*, *S. typhimurium*, and *C. pinatubonensis* (Brunet et al. 2020; Chakraborty et al. 2011; Li et al. 2022; Lin et al. 2017; Sana et al. 2012; Wang et al. 2019), in those systems, Fur mediates regulation by iron, and therefore its role in Mn^2+^-dependent T6SS expression represents a novel function. Although classical Fur is an iron-responsive regulator in most Gram-negative bacteria, recent studies have demonstrated that it is also involved in mediating manganese-dependent regulation of genes expression. For example, Fur in *Sinorhizobium meliloti* and *Rhizobium leguminosarum* mediates Mn^2+^ control of the *sitABCD* operon (Morrissey et al. 2004; Platero et al. 2004), and mediates both iron- and manganese-responsive regulation of *sitABCD* operon in *Agrobacterium tumefaciens* and the *yfeABCD* system in *Y. pestis (*Bearden and Perry 1999*;* Kitphati et al. 2007*)*. Our previous report has shown that Fur in *Y. pseudotuberculosis* regulates the aerobactin-mediated iron acquisition system in an iron concentration-dependent manner (Li et al. 2021). In this study, we further showed that Fur controls the T6SS4-mediated Mn^2+^ transport system in response to manganese (Figs. 2 and 3). Therefore, Fur in *Y. pseudotuberculosis* acts as not only an iron-dependent but also a manganese-responsive regulator, playing crucial roles in the maintenance of iron and manganese homeostasis.

Metal ions are commonly found in all organisms, and are involved in many crucial biological processes that are indispensable for survival in the environment or in their infected host (Porcheron et al. 2013). Bacteria have evolved many effective strategies to acquire essential metal ions. T6SS is a newly discovered mode of transport for metal ions such as zinc, manganese, iron, copper, and molybdenum; this finding greatly expands our understanding on the sophistication of bacterial metal ion acquisition systems (Lin et al. 2021; Yang et al. 2021). Our previous study found that *Y. pseudotuberculosis* T6SS4 secrets a Mn^2+^-binding micropeptide TssS for Mn^2+^ acquisition from the external or host environment, which contributes to manganese homeostasis as observed by the lower Mn^2+^ levels in the Δ*clpV4* mutant strain than in the WT (Zhu et al. 2021). Here we focused on the specific regulatory mechanism of T6SS4-mediated Mn^2+^ transport. Notably, our data showed that T6SS is induced by low Mn^2+^ but repressed by abundant exogenous Mn^2+^ due to the manganese-responsive Fur regulator (Fig. 3C). Thus, the T6SS4-mediated Mn^2+^ transport system represents an emergency manganese-acquisition strategy for *Y. pseudotuberculosis* under manganese deficient conditions.

Manganese, as an essential micronutrient transition metal, is required for mitigating oxidative stress by serving as a cofactor for ROS-detoxifying enzymes such as SodA and KatN or forming of antioxidants (Aguirre and Culotta 2012; Barnese et al. 2012). Therefore, we further investigated the role of the T6SS4-mediated Mn^2+^ transport system in resistance to oxidative stress; T6SS4 expression was significantly induced by H_2_O_2_, which might be due to the observation that the Fur-mediated repression of T6SS4 was substantially eliminated upon H_2_O_2_ challenge (Fig. 4A and B). Consistently, all mutants lacking conserved T6SS4 structural genes were more sensitive to oxidative stress than the WT (Fig. 4C and D). Moreover, exogenous Mn^2+^ markedly increased the survival rate of the WT and the complemented Δ*clpV4*(*clpV4*) strains under oxidative challenge, whereas the protective effect of exogenous Mn^2+^ was largely abolished in the Δ*clpV4* mutant (Fig. 4E and F). Based on these observations, we conclude that T6SS4 functions to combat oxidative stress by importing exogenous Mn^2+^ in *Y. pseudotuberculosis*.

As a versatile weapon of Gram-negative bacteria, T6SS plays important roles not only in bacteria competition and environmental adaption, but also in host infection (Cho et al. 2021; Lin et al. 2021; Monjaras Feria and Valvano 2020; Mougous et al. 2006). In this study, we demonstrated that T6SS4 contributes to the virulence and enhances the fitness of *Y. pseudotuberculosis* in the mouse infection model, which may help the bacteria occupy ecological niches within the host gut (Figs. 5 and 6). Noteworthily, our previous studies have reported that T6SS4 from *Y. pseudotuberculosis* is involved in the uptake of Zn^2+^ and Mn^2+^ (Wang et al. 2015; Zhu et al. 2021). Moreover, among pathogenic bacteria, a crucial benefit of Zn^2+^ and Mn^2+^ acquisition systems is to compete for essential metal ions to fight against host immunity (Becker and Skaar 2014; Diaz-Ochoa et al. 2016; Kehl-Fie et al. 2011; Radin et al. 2019). Therefore, it is not surprising that T6SS4-mediated Zn^2+^ and Mn^2+^ acquisition may offer bacteria an advantage in pathogenesis by evading host response.

In conclusion, our results reveal that T6SS4 from *Y. pseudotuberculosis* is directly regulated by Mn^2+^ via Fur, which combats oxidative stress and enhances bacterial virulence and fitness. We identify a new regulator Fur of the T6SS4-mediated Mn^2+^ acquisition system in *Y. pseudotuberculosis*, providing a new perspective on the regulatory mechanisms and functions of T6SS.

## Materials and methods

### Mice

C57BL/6 mice were purchased from the Animal Center of Xi’an JiaoTong University (SCXK: Shan 2012–003, Xi’an, China). All mouse experimental procedures were performed in accordance with the Regulations for the Administration of Affairs Concerning Experimental Animals approved by the State Council of the People’s Republic of China.

### Bacterial strains and growth conditions

Bacterial strains and plasmids used in this study are listed in Supplementary Table S1. *E. coli* strains were grown in Luria Bertani (LB) broth with appropriate antibiotics at 37 °C. The *Y. pseudotuberculosis* (YPIII) strains were cultured in Yersinia-Luria-Bertani (YLB) broth (1% tryptone, 0.5% yeast extract, 0.5% NaCl) or M9 minimal medium (Na_2_HPO_4_, 6 g L^− 1^; KH_2_PO_4_, 3 g L^− 1^; NaCl, 0.5 g L^− 1^; NH_4_Cl, 1 g L^− 1^; MgSO_4_, 2 mM; CaCl_2_, 0.1 mM; glucose 0.2%, pH 7.0) at 26 °C with appropriate antibiotics when necessary. All chemicals were of Analytical Reagent Grade purity or higher. The *Y. pseudotuberculosis* WT was the parent of all derivatives used in this study. In-frame deletions were generated as described previously (Xu et al. 2014b). Cellular growth was monitored based on the optical density (OD) at 600 nm. Antibiotics were added at the following concentrations: nalidixic acid, 20 μg mL^− 1^; kanamycin, 50 μg mL^− 1^; chloramphenicol, 20 μg mL^− 1^; tetracycline, 10 μg mL^− 1^.

### Plasmid construction

Primers used in this study are listed in Supplementary Table S2, respectively. The plasmid pDM4-Δ*fur* (*ypk_2991*) was used to construct the Δ*fur* in-frame deletion mutant of *Y. pseudotuberculosis*. A 626-bp upstream fragment and a 572-bp downstream fragment of *fur* were amplified using the primer pairs *fur-*1F*-*BglII/*fur*-1R and *fur*-2F/*fur*-2R-SalI, respectively. The upstream and downstream PCR fragments were ligated by overlapping PCR. The resulting PCR products were digested with BglII/SalI and inserted into the BglII/SalI sites of pDM4 to produce pDM4-Δ*fur*. The knock-out plasmids pDM4-Δ*vgrG4* (*ypk_3558*) and pDM4-Δ*katE* (*ypk_2855*) were constructed in a similar method by using primers list in Supplementary Table S2.

To complement the Δ*fur* mutant, primers *fur-*F-BamHI/*fur-*R-SalI were used to amplify the *fur* gene from the *Y. pseudotuberculosis* genome DNA. The PCR product of *fur* was digested with BamHI/SalI and inserted into the BamHI/SalI sites of pKT100 to produce pKT100-*fur*. The complementation plasmids pKT100-*vgrG4*, pKT100-*icmF4* (*ypk_3550*), pKT100-*sodB* (*ypk_1863*), pKT100-*katG* (*ypk_3388*), and pKT100-*katE* were constructed in a similar method by using primers list in Supplementary Table S2.

To express His_6_-tagged Fur, plasmid pET28a-*fur* was constructed. Briefly, primers *fur-*F-BamHI and *fur-*R-SalI were used to amplify the *fur* gene fragment from the *Y. pseudotuberculosis* genome. The PCR product of *fur* was digested with BamHI/SalI and inserted into the BamHI/SalI sites of pET28a to generate pET28a-*fur*.

For complementation, complementary plasmids pKT100-*fur*, pKT100-*vgrG4*, pKT100-*icmF4*, pKT100-*sodB*, pKT100-*katG*, and pKT100-*katE* were introduced into respective mutants by electroporation. The integrity of the insert in all constructs was confirmed by DNA sequencing.

### Overexpression and purification of recombinant protein

To express and purify soluble His_6_-tagged recombinant proteins, the plasmid pET28a-*fur* was transformed into BL21(DE3). Bacteria were cultured at 37 °C in LB medium to an OD_600_ of 0.5, shifted to 24 °C and induced with 0.2 mM IPTG, and then cultivated for an additional 12 h at 24 °C. Harvested cells were disrupted by sonication and proteins were purified with the His•Bind Ni-NTA resin (Novagen, Madison, WI) according to manufacturer’s instructions. Eluted recombinant proteins were dialyzed against buffer (50 mM Tris, 137 mM NaCl, 10% glycerol, pH 7.5) at 4 °C. The resulting proteins were stored at − 80 °C until use. Protein concentrations were determined using the Bradford assay according to the manufacturer’s instructions (Bio-Rad, Hercules, CA) with bovine serum albumin as standard.

### Metal-free apo-Fur preparation and metal ion binding assay

Metal ion binding assay was performed as previously described (Si et al. 2017b). Briefly, for removing all binding ions, purified Fur protein (300 μM) was added to the solution containing 50 mM Tris, 25 mM diethylene triamine pentaacetic acid, and 10% glycerol at pH 7.4. After incubation for 1 h on ice, the protein solution was dialyzed three times with buffer (50 mM Tris, 10% glycerol, pH 8.0) at 4 °C. For reconstitution with metal ions, the resulting apo-Fur protein (100 μM) was added to 500 μM of the desired divalent-metal ions (Fe^2+^ or Mn^2+^) and incubated on ice for 30 min, with Milli-Q water for preparing ions solution as the control. These solutions were dialyzed again to remove unbound metal ions and the metal ions bound to the protein were analyzed using atomic absorption spectroscopy (ZEEnit 650P; Analytik Jena, Jena, Germany).

### Determination of intracellular ion contents

Intracellular ion content was determined as described previously ( (Wang et al. 2015) b). Briefly, cells were grown in YLB medium until mid-exponential phase. After 20 mL of culture solution was collected and washed with M9 for two times, the cell pellet weight was measured and the bacterial cell was chemically lysed using Bugbuster (Novagen, Madison, WI) for 12 h. Total protein for each sample was measured by using NanoDrop ND-1000 spectrophotometer. Each sample was diluted 10-fold in 2% molecular grade nitric acid to a total volume of 5 mL at a slow setting for 12 h. Samples were analyzed by inductively coupled plasma mass spectrometry (ICP-MS), and the result was corrected using the appropriate buffers for reference and dilution factors.

### Electrophoretic Mobility Shift Assay (EMSA)

Electrophoretic mobility shift assay was performed by Zhang and colleagues (Zhang et al. 2013). P_T6SS4_ fragment was amplified from the *Y. pseudotuberculosis* genome with primers PT6SS4-F and PT6SS4-R. Increasing concentrations of purified apo-His_6_-Fur were incubated with 4 ng DNA probes in EMSA buffer (20 mM Tris, pH 7.4, 100 mM NaCl, 1 mM dithiothreitol, 10% glycerol) with or without of 100 μM Fe^2+^ or 100 μM Mn^2+^. After incubation for 20 min at room temperature, the binding reaction mixture was subjected to electrophoresis on a 6% native polyacrylamide gel containing 5% glycerol in 0.5 × TBE (Tris-borate-EDTA) electrophoresis buffer, and the DNA probe was detected using SYBR Green. As negative controls, a 350 bp DNA fragment amplified from the *Reut*_*B4659* coding region of the *C. pinatubonensis* JMP134 genomic DNA using primers control-F/control-R (Supplementary Table S2) and irrelevant protein (BSA) were included in the binding assay.

### Construction of chromosomal fusion reporter strains and β-galactosidase assays

The *lacZ* fusion reporter vectors pDM4-P_*T6SS1*_*::lacZ*, pDM4-P_*T6SS2*_*::lacZ*, pDM4-P_*T6SS3*_*::lacZ* and pDM4-P_*T6SS4*_*::lacZ* were transformed into *E. coli* S17–1 *λ pir* and mated with *Y. pseudotuberculosis* strains as described previously (Zhang et al. 2013). The *lacZ* fusion reporter strains were grown to stationary phase in YLB at pH 7.0 under 26 °C unless otherwise specified, and β-galactosidase activity was assayed using ONPG (o-Nitrophenyl β-D-galactopyranoside) as the substrate. These assays were performed in triplicate at least three times, and error bars represent standard deviations.

### Bacterial survival assays

Mid-exponential phase *Y. pseudotuberculosis* strains grown in YLB medium were collected, washed, and diluted 50-fold into M9 medium, and treated with CHP (0.5 mM) or H_2_O_2_ (1.0 mM), respectively, at 26 °C for 40 min. After treatment, the cultures were serially diluted and plated onto YLB agar plates, and colonies were counted after 36 h growth at 26 °C. Percentage survival was calculated by dividing the number of CFU of stressed cells by the number of CFU of cells without stress. All these assays were performed in triplicate at least three times.

### Protein secretion assay

Secretion assay for Hcp4 was performed according to described methods (Wang et al. 2015). Briefly, strains were inoculated into 100 mL YLB and incubated with continuous shaking until OD_600_ reached 1.5 at 26 °C. One milliliter of culture was centrifuged and the cell pellet was resuspended in 100 μL SDS loding buffer; this whole-cell lysate sample was defined as Hcp4_cells_. Two hundred fifty milliliter of the culture was centrifuged, and the supernatant was filtered through a 0.22 μm filter (Millipore, MA, USA). The secreted proteins in the supernatant were collected by filtration over a nitrocellulose filter (BA85) (Whatman, Germany) for three times. The filter was soaked in 100 μL SDS sample buffer for 15 min at 65 °C to recover the proteins present, and the sample was defined as Hcp4_sup_. All samples were normalized to the OD_600_ of the culture and volume used in preparation.

### Western blot analysis

Samples were resolved by SDS-PAGE and transferred onto PVDF membranes (Millipore). The membrane was blocked with QuickBlock™ Blocking Buffer (Beyotime Biotechnology, Haimen, China) for 30 min at room temperature and incubated with primary antibodies at 4 °C overnight: anti-VSV-G (Santa Cruz Biotechnology, USA), 1:500; anti-ICDH, 1:6000; The membrane was washed three times in TBST buffer (50 mM Tris, 150 mM NaCl, 0.05% Tween 20, pH 7.4), and incubated with 1:5000 dilution of horseradish peroxidase-conjugated secondary antibodies (Beyotime Biotechnology, Haimen, China) for 1 h. Signals were detected using the ECL kit (Invitrogen) following the manufacturer’s specified protocol.

### qRT-PCR

Bacteria cells were harvested during the mid-exponential phase and RNA was extracted using the RNA prep Pure Cell/Bacteria Kit and treated with RNase-free DNase (TIANGEN, Beijing, China). The purity and concentration of the RNA were determined by gel electrophoresis and spectrophotometer (NanoDrop, Thermo Scientific). First-strand cDNA was reverse transcribed from 1 μg of total RNA with the TransScript First-Strand cDNA Synthesis SuperMix (TransGen Biotech, Beijing, China). Quantitative real-time PCR (qRT-PCR) was performed in CFX96 Real-Time PCR Detection System (Bio-Rad, USA) with TransStart Green qPCR SuperMix (TransGen Biotech, Beijing, China). For all primer sets (Supplementary Table S2), the following cycling parameters were used: 95 °C for 30 s followed by 40 cycles of 94 °C for 15 s, 50 °C for 30 s. For standardization of results, the relative abundance of 16S rRNA was used as the internal standard. All samples were analyzed in triplicate, and the expression of target genes was calculated as relative fold values using the 2^-ΔΔCT^ method. These assays were performed in triplicate at least three times, and error bars represent standard error of the mean.

### RNA-seq experiment

Total RNA was extracted from *Y. pseudotuberculosis* WT and the Δ*fur* mutant grown in YLB at 26 °C with shaking (220 rpm) to a final optical density of approximately 1.6, using bacteria total RNA isolation kit (TIANGEN, Beijing, China). RNA degradation and contamination were monitored on 1% agarose gels; RNA purity was checked using the NanoPhotometer spectrophotometer (IMPLEN, CA, USA) and RNA integrity was assessed using the Bioanalyzer 2100 system (Agilent Technologies, CA, USA). A total of 3 μg RNA per sample was used as input material in RNA sample preparations for subsequent cDNA library construction. All samples had RIN values above 7.0. Sequencing libraries were generated using Illumina HiSeq™ 2000 RNA Sample Preparation Kit (Illumina, San Diego, USA) following manufacturer’s recommendations, and four index codes were added to attribute sequences to each sample. Differential expression analysis was performed using the NOIseq method (Sonia Tarazona 2100). *P*-values were adjusted using the Benjamini & Hochberg method. Corrected *P*-value of 0.05 and log_2_(fold change) of 0.8 were set as the threshold for significantly differential expression. Gene Ontology (GO) enrichment analysis of differentially expressed genes was implemented by the GOseq R package, in which gene length bias was corrected. GO terms with corrected *P*-value less than 0.05 were considered significantly enriched by differential expressed genes. The data have been deposited under bioProject accession number PRJNA632467.

### Mouse infections

Mid-exponential phase *Y. pseudotuberculosis* strains grown in YLB medium at 26 °C, washed twice in sterilized PBS and used for orogastric infection of 6–8 weeks old female C57BL/6 mice using a ball-tipped feeding needle (Schweer et al. 2013). For survival assays, 1 × 10^9^ bacteria of each strain were applied to different groups of mice, and the survival rate of the mice was determined by monitoring the survival daily for 21 days. For the analysis of the bacterial load in the feces, the fecal was sampled from individual living mice at specific time points, weighed and homogenized in PBS. For the analysis of the bacterial load in the cecum and small intestine, mice were sacrificed by carbon dioxide asphyxiation followed with cervical dislocation at specific time points after infection, the tissue was weighed and homogenized in PBS, and serial dilutions of the homogenates were plated on YLB plates with 20 μg mL^− 1^ nalidixic acid. The CFU was counted and given as CFU per gram of organ/tissue. In mice co-infected with two strains of *Y. pseudotuberculosis*, the index was calculated by dividing the output ratio (CFU of WT divided by CFU of the mutant) by the input ratio (CFU of WT divided by CFU of the mutant).

### Histology

For hematoxylin and eosin (H&E) staining, the cecum and liver of mice positively tested for *Yersinia* strains were excised at the indicated time points, fixed in 4% formalin for 24 to 48 h and embedded in paraffin. Three micrometer sections were stained with H&E. For each group 3 to 5 mice were blindly analyzed with light-microscopy by a histopathologist.

### Bioinformatics analyses

Sequence alignment and database searches were carried out using the BLAST server of the National Center for Biotechnology Information (NCBI) website (https://www.ncbi.nlm.nih.gov/) and visualized by using DNAMAN software.

### Statistical analysis

Experimental data analyzed for significance were performed by using GraphPad Prism 8 (GraphPad Software, San Diego California USA). *P* values for mice survival were calculated using Log-rank (Mantel-Cox) test. *P* values for bacterial CFU in mouse tissues were calculated using Mann-Whitney test (I). Statistical analyses for the rest of the assays were performed using unpaired two-tailed Student’s t-test. Error bars represent ± SEM. *P < 0.05; **P < 0.01; ***P < 0.001.

## Supplementary Information

**Additional file 1: Fig. S1.** Analysis amino acid sequence of Fur in *Y. pseudotuberculosis*. **Fig. S2.** Gene organization of T6SS gene clusters in *Y. pseudotuberculosis*. **Fig. S3.** Transcriptional regulation analysis of T6SS1, T6SS2, and T6SS3 by Fur in *Y. pseudotuberculosis*. **Fig. S4.** Growth curves of the *Y. pseudotuberculosis* WT, Δ*fur* mutant, and the complemented strain Δ*fur*(*fur*). **Fig. S5.** Fur binds directly to the T6SS4 promoter with high affinity in an Mn^2+^-dependent manner. **Fig. S6.** Purified recombinant Fur was analyzed by 12% SDS-PAGE. **Table 1.** Bacterial strains and plasmids. **Table 2.** Primers used in this study.

## Data Availability

All datasets generated for this study are included in the article/Supplementary Information.

## Abbreviations

T6SS: Type VI secretion system
Mn: Manganese
Fe: Iron
Zn: Zinc
Fur: Ferric uptake regulator
OxyR: Oxidative stress regulator
Zur: Zinc uptake regulator
RNA-seq: RNA sequencing
OMVs: Outer membrane vesicles
DEGs: Differentially expressed genes
WT: Wild type
qRT-PCR: Quantitative real time polymerase chain reaction
YLB: Yersinia–Luria–Bertani
EMSA: Electrophoretic mobility shift assay
ICP-MS: Inductively coupled plasmon resonance atomic absorption spectrometry
EDDHA: Ethylenediamine-*N,N*′-bis(2-hydroxyphenylacetic acid
H_2_O_2_: Hydrogen peroxide
CHP: Cumene hydroperoxide
H&E: Hematoxylin and eosin
CFU: Colony-forming unit
