# Supplementary material for: A Fur-regulated type VI secretion system contributes to oxidative stress resistance and virulence in Yersinia pseudotuberculosis
Source: Stress Biol. 2023 Jan 6;3(1):2. doi: 10.1007/s44154-022-00081-y (PMC10441874; doi:10.1007/s44154-022-00081-y)
Supplement: Supplementary file 1 — Additional file 1: Fig. S1. Analysis amino acid sequence of Fur in Y. pseudotuberculosis. Fig. S2. Gene organization of T6SS gene clusters in Y. pseudotuberculosis. Fig. S3. Transcriptional regulation analysis of T6SS1, T6SS2, and T6SS3 by Fur in Y. pseudotuberculosis. Fig. S4. Growth curves of the Y. pseudotuberculosis WT, Δfur mutant, and the complemented strain Δfur(fur). Fig. S5. Fur binds directly to the T6SS4 promoter with high affinity in an Mn2+-dependent manner. Fig. S6. Purified recombinant Fur was analyzed by 12% SDS-PAGE. Table 1. Bacterial strains and plasmids. Table 2. Primers used in this study. [file 44154_2022_81_MOESM1_ESM.docx]

**Supplementary Information**

**A Fur-regulated type VI secretion system contributes to**

**oxidative stress resistance and** **virulence in *Yersinia pseudotuberculosis***

**This PDF file includes:**

**Supplementary Figures 1-6**

**Supplementary Tables 1-2**

**Supplementary References**


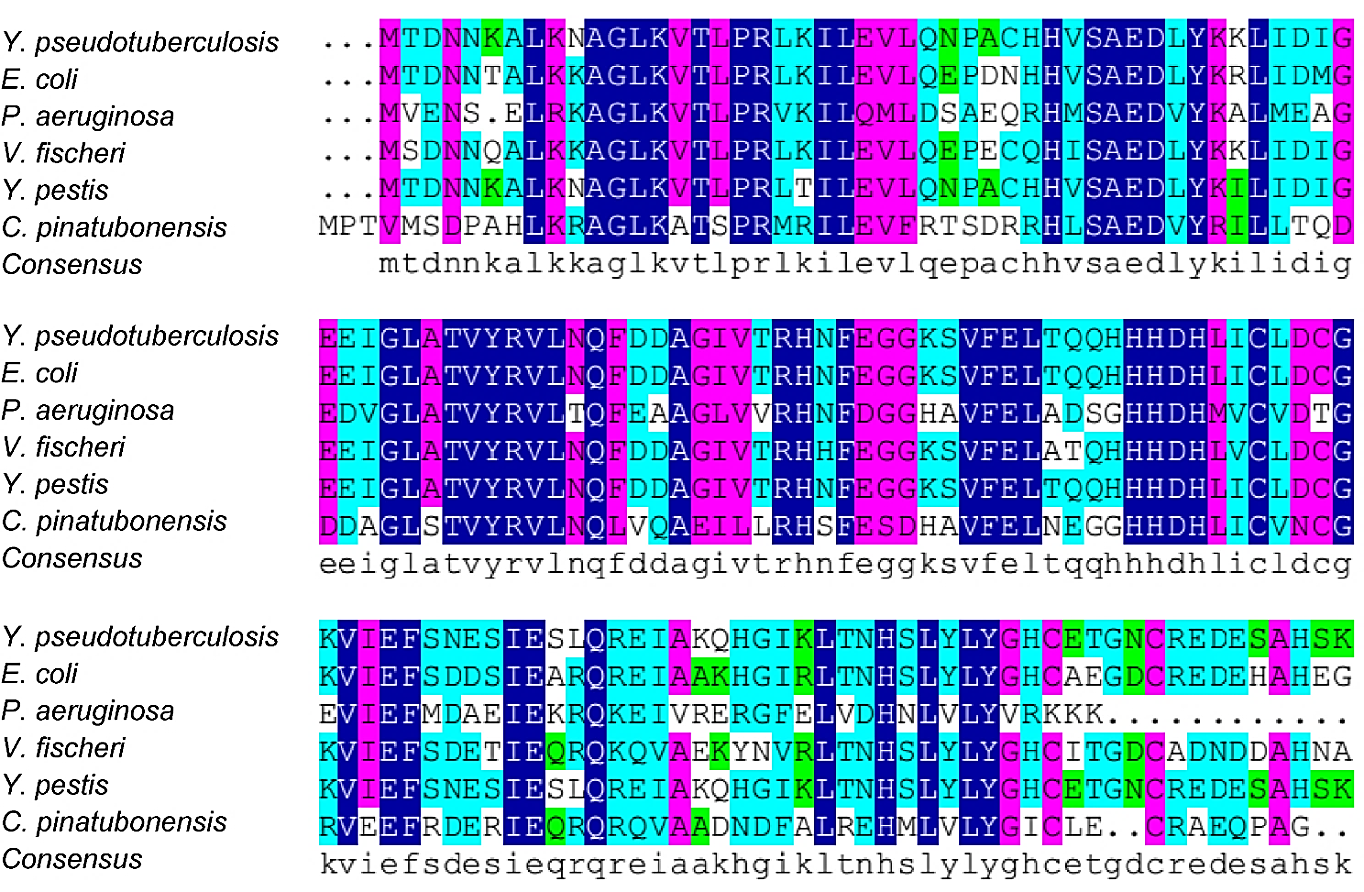


# Fig. S1 Analysis amino acid sequence of Fur in *Y. pseudotuberculosis*.

Comparison of Fur amino acid sequences from different species. The accession numbers of the *fur* genes from different species are as follows: *Yersinia pseudotuberculosis* YPIII (*ypk_2991*), *Escherichia coli* K-12 (*b0683*), *Pseudomonas aeruginosa* PAO1 (*PA4764*), *Vibrio fischeri* ES114 (*VF_0810*), *Yersinia* *pestis* 91001 (*YP_1081*), *Cupriavidus* *pinatubonensis* JMP134 (*Reut_B5530*).


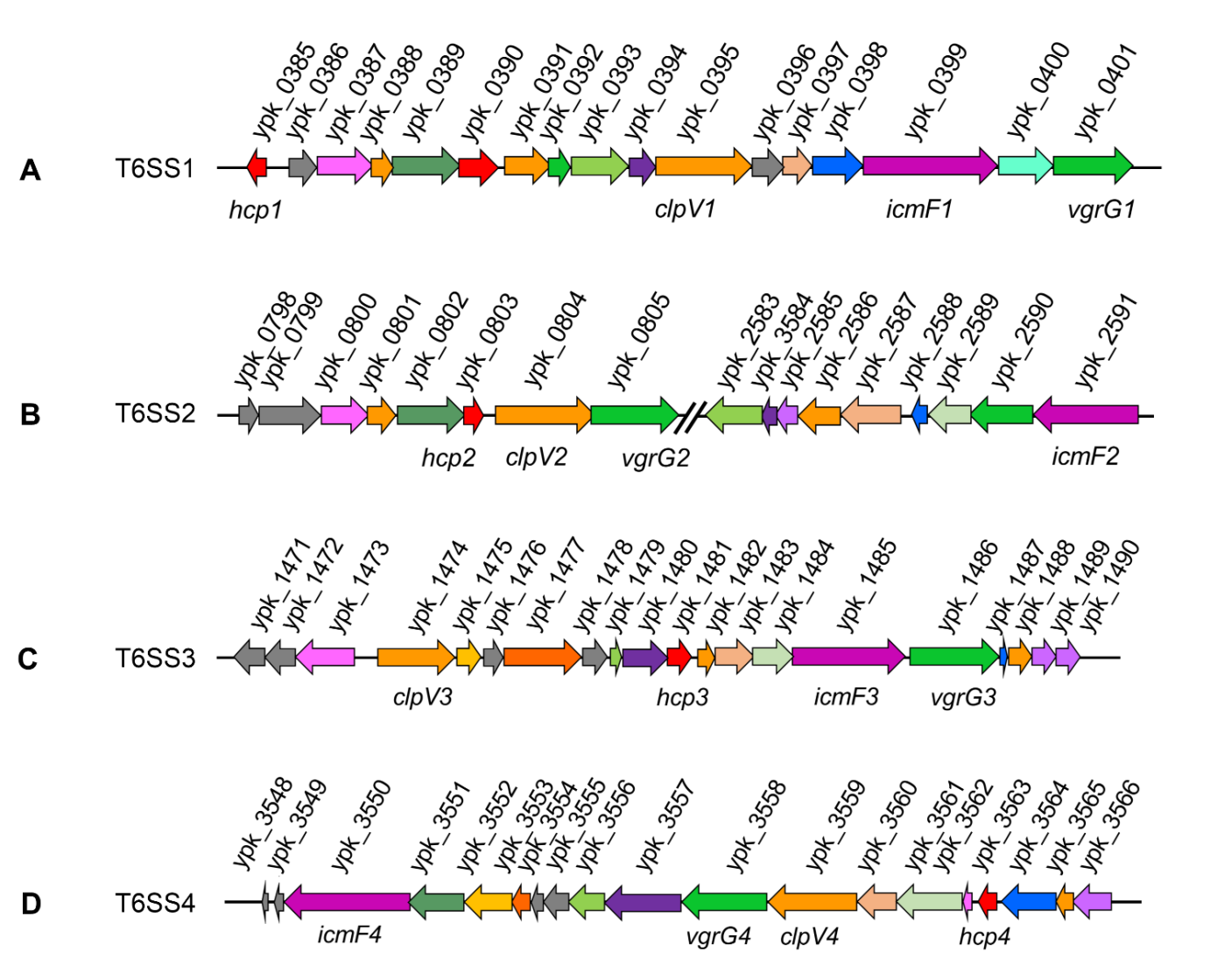


# Fig. S2 Gene organization of T6SS gene clusters in *Y. pseudotuberculosis*.

Arrows represent the location and direction of transcription of T6SS genes. Above each gene is the locus number.


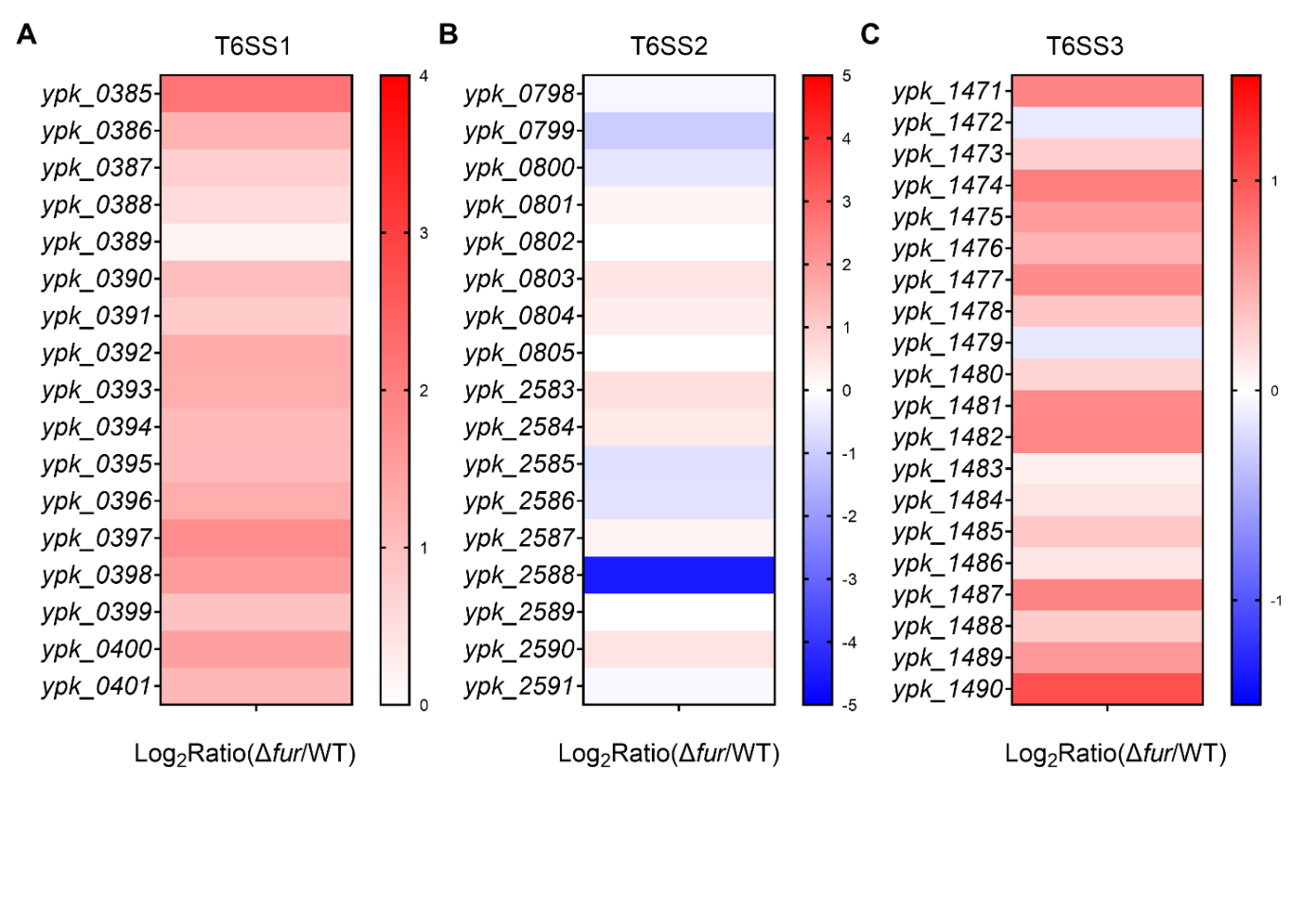


# Fig. S3 Transcriptional regulation analysis of T6SS1, T6SS2, and T6SS3 by Fur in *Y. pseudotuberculosis*.

All genes in the T6SS1 (A), T6SS2 (B), and T6SS3 (C) transcribed in the *Y. pseudotuberculosis* Δ*fur* mutant compared with those in the WT were detected by RNA-seq data analysis.


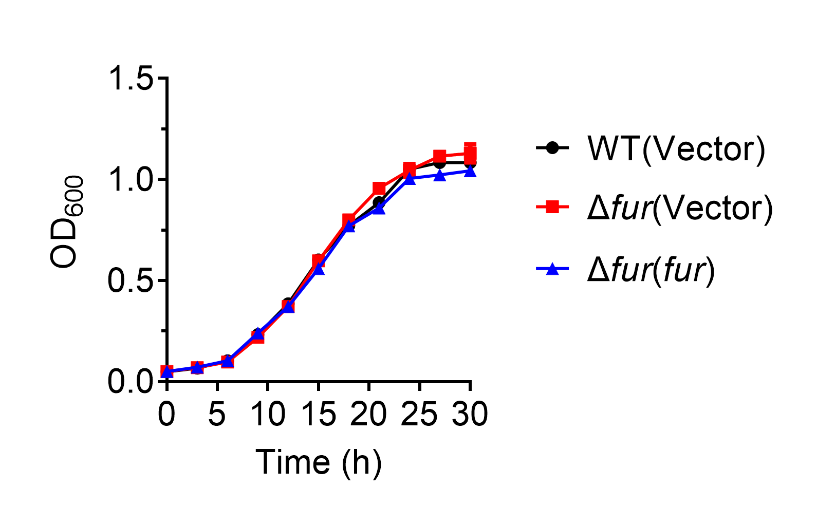


# Fig. S4 Growth curves of the *Y. pseudotuberculosis* WT, Δ*fur* mutant, and the complemented strain Δ*fur*(*fur*).

Relevant *Y. pseudotuberculosis* strains grown overnight in 5 ml of YLB medium were diluted 1:100 in fresh YLB medium. The growth of the cultures was monitored by measuring OD_600_ at indicated time points.

Data represent the mean ± SEM of three biological replicates, each of which was performed with three technical replicates.


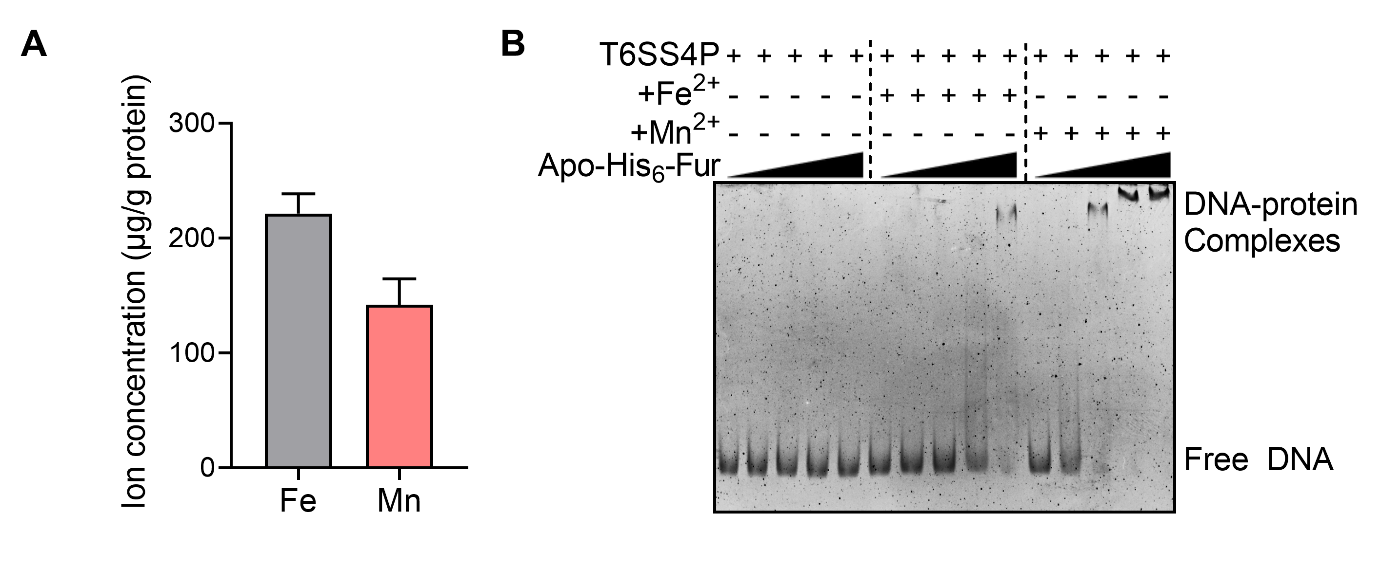


# Fig. S5 Fur binds directly to the T6SS4 promoter with high affinity in an Mn^2+^-dependent manner

(**A**) The binding of divalent ions by metal-free apo-His_6_-Fur was detected by atomic adsorption spectrometry. (**B**) Effects of Mn^2+^ and Fe^2+^ on binding of *Y. pseudotuberculosis* Fur to the promoter. EMSA was performed to analyze the interaction between metal-free apo-His_6_-Fur and the T6SS4 promoter (*P_T6SS4_*) in the presence or absence of 100 µM Fe^2+^ or 100 µM Mn^2+^. Increasing amounts of Fur (0.12, 0.24,0.48, 0.96, and 1.92 μM) and 4 ng DNA fragment were used.


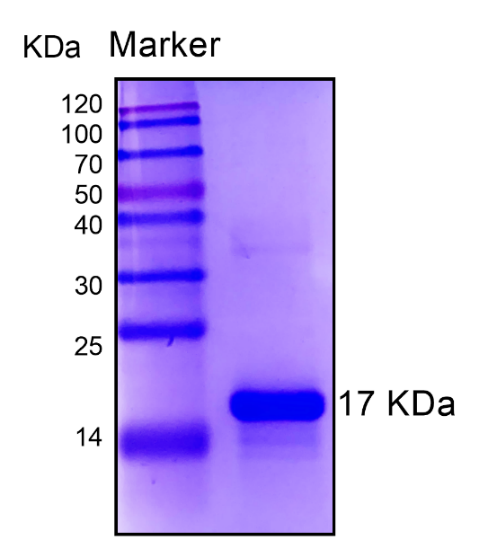


# Fig. S6 Purified recombinant Fur was analyzed by 12% SDS-PAGE.

**Table 1 Bacterial strains and plasmids.**

| **Strain or plasmid** | **Relevant characteristics** | **Reference** | |
| --- | --- | --- | --- |
| ***E. coli*** | | | |
| S17-1*λ pir* | *λ pir* lysogen of S17-1, *thi pro hsdR hsdM^+^ recA* RP4 -Tc::Mu-Km::Tn7 | ([Simon et al., 1983](#_ENREF_6)) | |
| BL21(DE3) | Host for expression vector pET28a | Novagen | |
| DH5α | FΦ80Δ*lacZ*ΔM15/Δ(*lacZYA-argF*)*U169recA1 endA1 hsdR17* | Novagen | |
| ***C. necator*** |  |  | |
| JMP134 | Wild-type *Cupriavidus pinatubonensis* JMP134 | ([Li et al., 2019](#_ENREF_3)) | |
| ***Y. pseudotuberculosis*** | | | |
| WT | Wild-type *Yersinia pseudotuberculosis* YPIII, Nal^r^ | ([Rosqvist et al., 1988](#_ENREF_5)) | |
| Δ*fur* | *fur* gene deleted in *Y. pseudotuberculosis*, Nal^r^ | This study | |
| Δ*clpV4* | *clpV4* gene deleted in *Y. pseudotuberculosis*, Nal^r^ | ([Zhang et al., 2013](#_ENREF_8)) | |
| Δ*vgrG4* | *vgrG4* gene deleted in *Y. pseudotuberculosis*, Nal^r^ | This study | |
| Δ*icmF4* | *icmF4* gene deleted in *Y. pseudotuberculosis*, Nal^r^ | ([Wang et al., 2015](#_ENREF_7)) | |
| Δ*sodB* | *sodB* gene deleted in *Y. pseudotuberculosis*, Nal^r^ | ([Wang et al., 2015](#_ENREF_7)) | |
| Δ*katG* | *katG* gene deleted in *Y. pseudotuberculosis*, Nal^r^ | ([Wang et al., 2015](#_ENREF_7)) | |
| Δ*katE* | *katE* gene deleted in *Y. pseudotuberculosis*, Nal^r^ | This study | |
| WT(Vector) | WT(Vector) containing pKT100, Nal^r^, Km^r^ | This study | |
| Δ*fur*(Vector) | Δ*fur* containing pKT100, Nal^r^, Km^r^ | This study | |
| Δ*fur*(*fur*) | Δ*fur* containing pKT100-*fur*, Nal^r^, Km^r^ | This study | |
| Δ*clpV4*(Vector) | Δ*clpV4* containing pKT100, Nal^r^, Km^r^ | ([Zhang et al., 2013](#_ENREF_8)) | |
| Δ*clpV4*(*clpV4*) | Δ*clpV4* containing pKT100-*clpV4*, Nal^r^, Km^r^ | ([Zhang et al., 2013](#_ENREF_8)) | |
| Δ*vgrG4*(Vector) | Δ*vgrG4* containing pKT100, Nal^r^, Km^r^ | This study | |
| Δ*vgrG4*(*vgrG4*) | Δ*vgrG4* containing pKT100-*vgrG4*, Nal^r^, Km^r^ | This study | |
| Δ*icmF4*(Vector) | Δ*icmF4* containing pKT100, Nal^r^, Km^r^ | This study | |
| Δ*icmF4*(*icmF4*) | Δ*icmF4* containing pKT100-*icmF4*, Nal^r^, Km^r^ | This study | |
| Δ*sodB*(Vector) | Δ*sodB* containing pKT100, Nal^r^, Km^r^ | This study | |
| Δ*sodB*(*sodB*) | Δ*sodB* containing pKT100-*sodB*, Nal^r^, Km^r^ | This study | |
| Δ*katG*(Vector) | Δ*katG* containing pKT100, Nal^r^, Km^r^ | This study | |
| Δ*katG*(*katG*) | Δ*katG* containing pKT100-*katG*, Nal^r^, Km^r^ | This study | |
| Δ*katE*(Vector) | Δ*katE* containing pKT100, Nal^r^, Km^r^ | This study | |
| Δ*katE*(*katE*) | Δ*katE* containing pKT100-*katE*, Nal^r^, Km^r^ | This study | |
| **Plasmid** |  |  | |
| pKT100 | Cloning vector, p15A replicon, Km^r^ | ([Hu et al., 2009](#_ENREF_2)) | |
| pKT100-*fur* | *fur* under the control of chloramphenicol resistance gene promoter in plasmid pKT100, Km^r^ | This study | |
| pKT100-*vgrG4* | *vgrG4* under the control of chloramphenicol resistance gene promoter in plasmid pKT100, Km^r^ | This study | |
| pKT100-*icmF4* | *icmF4* under the control of chloramphenicol resistance gene promoter in plasmid pKT100, Km^r^ | This study | |
| pKT100-*sodB* | *sodB* under the control of chloramphenicol resistance gene promoter in plasmid pKT100, Km^r^ | This study | |
| pKT100-*katG* | *katG* under the control of chloramphenicol resistance gene promoter in plasmid pKT100, Km^r^ | This study | |
| pKT100-*katE* | *katE* under the control of chloramphenicol resistance gene promoter in plasmid pKT100, Km^r^ | This study | |
| pET28a | Expression vector with N-terminal hexahistidine affinity tag, Km^r^ | Novagen | |
| pET28a-*fur* | pET28a carrying *fur* coding region of, Km^r^ | This study | |
| pDM4 | Suicide vector, *mob*RK2, *ori*R6K, *pir*, *sacB*, Cm^r^ | | ([Milton et al., 1996](#_ENREF_4)) |
| pDM4-Δ*fur* | Construct used for in-frame deletion of *fur*, Cm^r^ | This study | |
| pDM4-Δ*vgrG4* | Construct used for in-frame deletion of *vgrG4*, Cm^r^ | This study | |
| pDM4-Δ*icmF4* | Construct used for in-frame deletion of *icmF4*, Cm^r^ | ([Wang et al., 2015](#_ENREF_7)) | |
| pDM4-Δ*sodB* | Construct used for in-frame deletion of *sodB*, Cm^r^ | ([Wang et al., 2015](#_ENREF_7)) | |
| pDM4-Δ*katG* | Construct used for in-frame deletion of *katG*, Cm^r^ | ([Wang et al., 2015](#_ENREF_7)) | |
| pDM4-Δ*katE* | Construct used for in-frame deletion of *katE*, Cm^r^ | This study | |
| pDM4-*T6SS1p*::*lacZ* | For *T6SS1* promoter fusion to *Y. pseudotuberculosis*, Cm^r^ | ([Zhang et al., 2013](#_ENREF_8)) | |
| pDM4-*T6SS2p*::*lacZ* | For *T6SS2* promoter fusion to *Y. pseudotuberculosis*, Cm^r^ | ([Zhang et al., 2013](#_ENREF_8)) | |
| pDM4-*T6SS3p*::*lacZ* | For *T6SS3* promoter fusion to *Y. pseudotuberculosis*, Cm^r^ | ([Zhang et al., 2013](#_ENREF_8)) | |
| pDM4-*T6SS4p*::*lacZ* | For *T6SS4* promoter fusion to *Y. pseudotuberculosis*, Cm^r^ | ([Zhang et al., 2013](#_ENREF_8)) | |
| pME6032 | Shuttle vector, Tc^r^ | ([Heeb et al., 2002](#_ENREF_1)) | |
| pME6032-*hcp4-vsvg* | pME6032 carrying *hcp4-vsvg* coding region, Tc^r^ | ([Wang et al., 2015](#_ENREF_7)) | |

*Nal^r^, Km^r^, Cm^r^, and Tc^r^ represent resistance to Naladixic acid, Kanamycin, Chloramphenicol, and Tetracycline at 20, 50, 20, and 10 μg mL^-1^, respectively.

**Table 2 Primers used in this study.**

| **Primers** | **5’-3’ sequence** | **Function** |
| --- | --- | --- |
| *fur-*1F*-*BglII | GGAAGATCTTTCTGTGATGCGATGGG | To generate pDM4-Δ*fur* |
| *fur-*1R | AAGGCTTTGTTGTTGTCAGT |  |
| *fur-*2F | **ACTGACAACAACAAAGCCTT**TGCCGTGAAGATGAGTC |  |
| *fur-*2R*-*SalI | CGCGTCGACGCTAAAGCGCAACCTACT |  |
| *vgrG4-*1F*-*BamHI | CGCGGATCCCCGAAGCGGTAAGACG | To generate pDM4-Δ*vgrG4* |
| *vgrG4-*1R | GGATGGATTCCCTGACACT |  |
| *vgrG4-*2F | **AGTGTCAGGGAATCCATCC**CAGTATTGGGACGGTAGG |  |
| *vgrG4-*2R*-*XhoI | CCGCTCGAGGGGCGCTGAAGGGTTA |  |
| *katE-*1F*-*BglII | GGAAGATCTCCACTGCTGCCGTCTT | To generate pDM4-Δ*katE* |
| *katE-*1R | CTCAGGGATCACTTCACG |  |
| *katE-*2F | **CGTGAAGTGATCCCTGAG**CCGTATTGCGGGTGAG |  |
| *katE-*2R*-*SalI | CGCGTCGACGATGAGCGGCTTGAGTG |  |
| *fur-*F*-*BamHI | CTGAGGATCCATGACTGACAACAACAAAGCCT | To generate pET28a-*fur*  pKT100-*fur* |
| *fur-*R*-*SalI | GTGCGTCGACTTATCTTTTACTGTGTGCAGACTCA |  |
| *icmF4-*F*-*BglII | GGAAGATCTATGATACGCAGAATGTTGAT | To generate pKT100-*icmF4* |
| *icmF4-*R*-*SalI | ACGCGTCGACTTAGAGATTCTTAGGACATGAG |  |
| *vgrG4-*F*-*BglII | GGAAGATCTATGCAATTAATTGAGATAGAG | To generate pKT100-*vgrG4* |
| *vgrG4-*R*-*SalI | ACGCGTCGACTCAGTTTTTGCTTTGCATG |  |
| *sodB-*F*-*BamHI | CGCGGATCCATGTCTTTTGAATTACCT | To generate pKT100-*sodB* |
| *sodB-*R*-*SalI | CGCGTCGACTCAGTCTAGGTTTTTCTC |  |
| *katG-*F*-*BamHI | CGCGGATCCATGTTAAAAAAAATCTTACCCGTAC | To generate pKT100-*katG* |
| *katG-*R*-*SalI | CGCGTCGACTTAGTTATTTTTTATATCAAAGCGA |  |
| *katE-*F*-*BamHI | CGCGGATCCATGAGCAAGAAGAAAGGATTAACCA | To generate pKT100-*katE* |
| *katE-*R*-*SalI | CGCGTCGACTTAATTCAGGCCAAGTGCTTTTTTC |  |
| PT6SS4-F | GTCCTCTTATTTTGGCTATT | EMSA |
| PT6SS4-R | ATTATTGTCCATCCGTTTTA |  |
| control-F | GCCATTGACGCCAACACTAC |  |
| control-R | TTCACGCGGTACGCACCCAT |  |
| Q16S-F | CTAGCGATTCCGACTTCAT | qRT-PCR |
| Q16S-R | CCCTTATCCTTTGTTGCC |  |
| Q*clpV4*-F | GGCGTCACCTTCTCCTATC |  |
| Q*clpV4*-R | TGAACCTCGCTGGTCTGT |  |
| Q*hcp4*-F | GTAACTGTCTGGTGTCCTCC |  |
| Q*hcp4*-R | CCATCAGGTTGCTGCTCT |  |
| Q*vgrG4*-F | AGGGAATCCATCCTACCA |  |
| Q*vgrG4*-R | AATTTGTCTTGCCGTTGC |  |
| Q*icmF4*-F | AGGCCAACCTCAGCAAG |  |
| Q*icmF4*-R | CCCAGAACGACCAAACATA |  |

Underlined sites Indicate restriction enzyme cutting sites added for cloning. Letters In boldface denote the annealing regions for overlap PCR.

**References:**

Heeb S, Blumer C, & Haas D (2002) Regulatory RNA as mediator in GacA/RsmA-dependent global control of exoproduct formation in *Pseudomonas fluorescens* CHA0. J Bacteriol 184(4):1046-1056. <https://doi.org/10.1128/jb.184.4.1046-1056.2002>

Hu Y, Lu P, Wang Y, Ding L, Atkinson S, & Chen S (2009) OmpR positively regulates urease expression to enhance acid survival of *Yersinia pseudotuberculosis*. Microbiology 155(Pt 8):2522-2531. <https://doi.org/10.1099/mic.0.028381-0>

Li C, Zhu L, Pan D, Li S, Xiao H, Zhang Z, Shen X, Wang Y, & Long M (2019) Siderophore-Mediated Iron Acquisition Enhances Resistance to Oxidative and Aromatic Compound Stress in *Cupriavidus necator* JMP134. Appl Environ Microbiol 85(1). <https://doi.org/10.1128/AEM.01938-18>

Milton DL, O'Toole R, Horstedt P, & Wolf-Watz H (1996) Flagellin A is essential for the virulence of *Vibrio anguillarum*. J Bacteriol 178(5):1310-1319. <https://doi.org/10.1128/jb.178.5.1310-1319.1996>

Rosqvist R, Skurnik M, & Wolf-Watz H (1988) Increased virulence of *Yersinia pseudotuberculosis* by two independent mutations. Nature 334(6182):522-524. <https://doi.org/10.1038/334522a0>

Simon R, Priefer UB, & Puhler A (1983) A broad host range mobilization system for *in vivo* genetic engineering: transposon mutagenesis in Gram negative bacteria. Nature Biotechnology 1(9):784-791.

Wang T, Si M, Song Y, Zhu W, Gao F, Wang Y, Zhang L, Zhang W, Wei G, Luo ZQ, & Shen X (2015) Type VI secretion system transports Zn^2+^ to combat multiple stresses and host immunity. PLoS Pathog 11(7):e1005020. <https://doi.org/10.1371/journal.ppat.1005020>

Zhang W, Wang Y, Song Y, Wang T, Xu S, Peng Z, Lin X, Zhang L, & Shen X (2013) A type VI secretion system regulated by OmpR in *Yersinia pseudotuberculosis* functions to maintain intracellular pH homeostasis. Environ Microbiol 15(2):557-569. <https://doi.org/10.1111/1462-2920.12005>
